# Lysolecithin reprogramming via LPCAT1 modulation restores endothelial function and prevents diabetes-associated dysmetabolism

**DOI:** 10.1186/s12933-025-03053-4

**Published:** 2026-01-18

**Authors:** Eduardo Maria Sommella, Concetta Iside, Paola Di Pietro, Fabrizio Merciai, Emanuela Salviati, Marina Sala, Angela Carmelita Abate, Antonio Damato, Massimiliano De Lucia, Eleonora Venturini, Valeria Prete, Francesca Picone, Paolo Poggio, Pasquale Mone, Michele Ciccarelli, Gaetano Santulli, Pietro Campiglia, Carmine Vecchione, Albino Carrizzo

**Affiliations:** 1 Department of Pharmacy, University of Salerno, SA 84084 Fisciano, Italy; 2 Department of Medicine, Surgery and Dentistry, “Scuola Medica Salernitana” University of Salerno, Baronissi, Italy; 3 Vascular Physiopathology Unit, IRCCS Neuromed, Pozzilli, Italy; 4 Centro Cardiologico Monzino IRCCS, Milan, Italy; 5 Department of Biomedical, Surgical, and Dental Sciences, University of Milan, Milan, Italy; 6 Department of Medicine and Health Sciences “Vincenzo Tiberio”, University of Molise, Campobasso, Italy; 7 Casa di Cura Montevergine, Mercogliano, Italy; 8 Department of Molecular, Cellular, and Biomedical Sciences, City University of New York, School of Medicine, Manhattan, NY USA; 9 International Translational Research and Medical Education (ITME) Consortium, Academic Research Unit, Naples, Italy

**Keywords:** Lipid metabolism, Diabetes, Endothelial function, Lysolecithin reprogramming

## Abstract

**Background:**

Dysregulation of lysophosphatidylcholines (LPCs) and phosphatidylcholines (PCs) is linked to endothelial dysfunction and impaired tissue repair. Nevertheless, the organ-specific modulation of lysolecithin remodeling in T2DM remains unexplored. Here, we investigate the LPC/PC remodeling dynamics in a T2DM model and propose a novel therapeutic approach using an orally bioavailable peptide (SP6) derived from Spirulina platensis.

**Methods:**

LPC/PC levels were analyzed by UHPLC-HRMS. Membrane fluidity, VEGF/API5, LPCAT1, VE-cadherin, and GLUT1 were evaluated by merocyanine assay, qPCR, immunoblotting, and immunofluorescence. In vivo, T2DM was induced by a high-fat diet and streptozotocin, and SP6 was orally administered. Tissue lipidomics, GLUTs expression, and insulin secretion were assessed, with the latter also spatially characterized in pancreatic tissue by MALDI-MS imaging.

**Results:**

High glucose induced LPC/PC imbalance, enhanced membrane fluidity, impaired VEGF/API5 expression, and hindered wound healing and VE-cadherin localization via LPCAT1 downregulation and subsequent impact on GLUT1 translocation. In vivo analysis of diabetic mice revealed a multi-organ influence of SP6 preserving LPCAT1 mRNA levels in pancreas, liver, skeletal muscle, and adipose tissue and a specific pattern of lysolecithin remodeling, with selective modulation of LPC 16:0, 18:0, and 20:4 in plasma. Finally, its effects in T2DM are mediated by preserving insulin secretion and glycemic control through increased ATP production.

**Conclusion:**

These findings reveal tissue-specific lysolecithin reprogramming in T2DM development and identify LPCAT1-mediated lysolecithin remodeling as a mechanism involved in T2DM-related endothelial and metabolic dysfunction. SP6 modulates lipid metabolism, vascular integrity, and glucose regulation at the transcript level, suggesting its potential as a new preventive treatment for T2DM and its complications.

**Graphical abstract:**

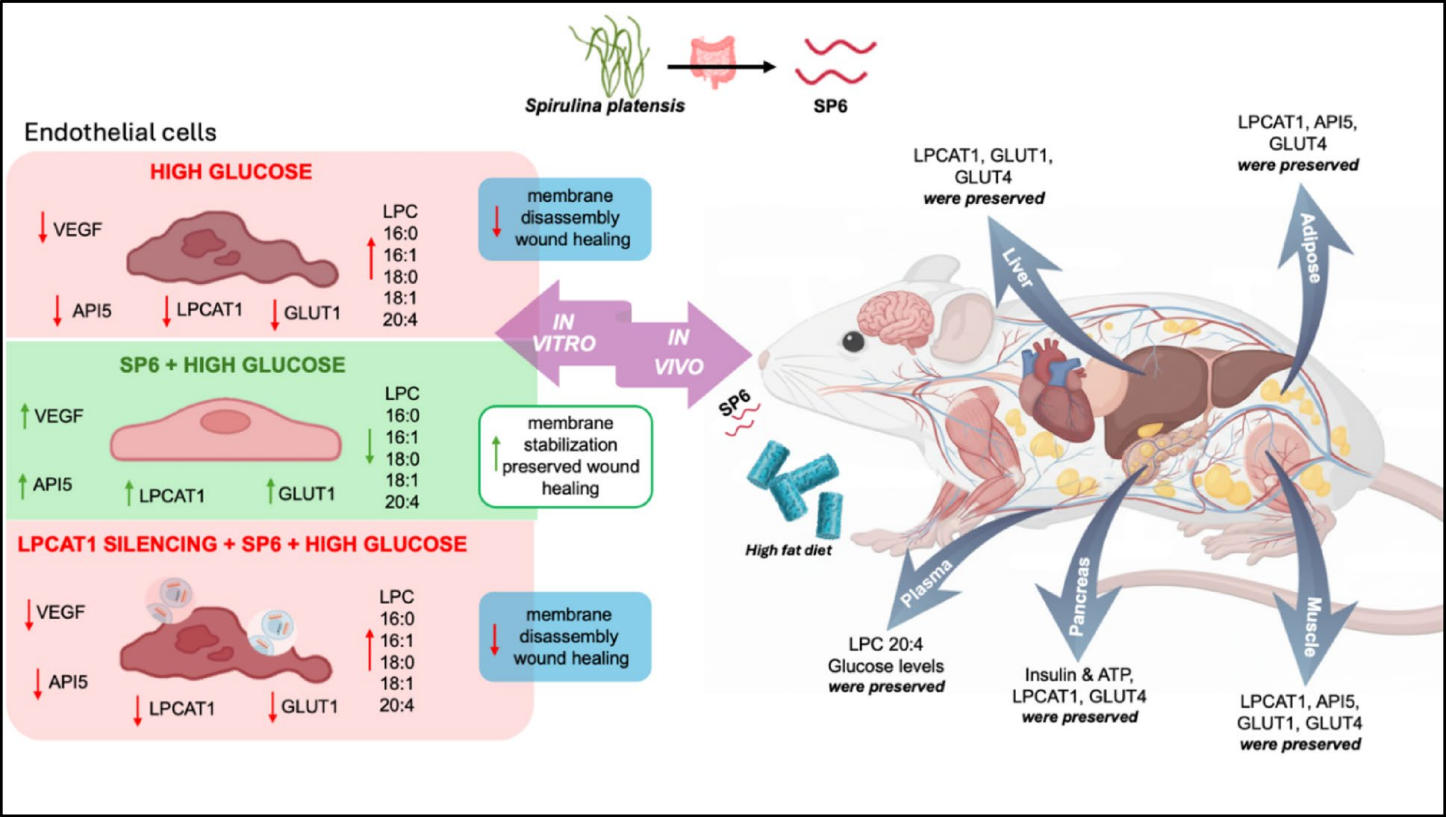

**Supplementary Information:**

The online version contains supplementary material available at 10.1186/s12933-025-03053-4.

## Research Insights

- **What is currently known about this topic?**
  Lipidomic studies in type 2 diabetes reveal disrupted phospholipid metabolism, however conflicting trends, especially in lysophosphatidylcholines (LPCs) and phosphatidylcholines (PCs) are reported
- **What is the key research question?**
  Can modulation of lysolecithin reprogramming machinery reduce diabetes onset and progression?
- **What is new?**
  T2DM is markedly affected by the impairment of lysolecithin machinery homeostasis. SP6 shows promise as a preventive strategy targeting both endothelial integrity and lysolecithin reprogramming machinery in T2DM acting on aberrant LPCAT1 modulation
- **How might this study influence clinical practice?**
  SP6 emerges as a promising preventive candidate for targeting aberrant lysolecithin remodeling pathways in diabetic patients

**Supplementary Information:**

The online version contains supplementary material available at 10.1186/s12933-025-03053-4.

## Introduction

The rising diabetes epidemic has revealed the limitations of a one-size-fits-all management approach [1, 2]. Despite medications, a healthy diet, regular physical activity, and lifestyle changes [3], both the number of cases and the prevalence of diabetes have been steadily increasing over the past few decades, with even greater growth predicted by 2045 [2]. Focus on stimulating the release of insulin from the pancreas or improving insulin-stimulated glucose uptake [1]. However, changes in lipid metabolism quickly occur in individuals with type 2 diabetes mellitus (T2DM) and might further impair endothelial integrity [2, 3] and incretin effects [4] that result from chronic hyperglycemia alone.

Recently, the analysis of lipid profiles in various diabetic conditions [5–8] has revealed an imbalance in phospholipid metabolism [9], particularly the dysregulation of lysophosphatidylcholines (LPCs) and phosphatidylcholines (PCs) [10–12], also called lysolecithins. Nevertheless, clinical lipidomic studies have been controversial. Some human studies revealed a widespread reduction in LPC species in plasma from obese T2DM individuals [13, 14]; on the other hand, other studies reported that LPC content was significantly higher in diabetic patients than in control healthy subjects [15]. However, this contrasting evidence has been obtained by evaluating lysolecithins only in human plasma, thereby leading to a misunderstanding of the complexity of the enzymatic cascade and the specific contributions of tissues to LPC and PC regulation. In this regard, the altered balance between LPC and PC has been implicated in the wound-healing mechanism. In particular, it impairs angiogenesis and affects proliferation, migration, and physiological cell function [16–18], which are hallmarks of insulin resistance and diabetic vascular complications [19]. So far, it is unknown whether the high glucose levels observed in diabetic conditions, per se, lead to specific alterations in subcellular compartments and multiorgan lipidome patterns contributing to the diabetic vascular phenotype.

In this study, we focused on elucidating the mechanism of action of a recently characterized *Spirulina platensis*-derived peptide, SP6 [20], under diabetic conditions, demonstrating its ability to preserve LPCAT1 expression, a key enzyme regulating the balance between LPCs and PCs. This regulation supports membrane stability and maintains essential factors for endothelial integrity. Using both cellular and animal models of T2DM, we show that SP6 exerts multifaceted protective effects by improving insulin secretion and glucose homeostasis across multiple metabolically relevant tissues. LPCAT1 emerges as a central mediator of SP6’s beneficial actions, highlighting the peptide as a promising therapeutic candidate to counteract vascular and metabolic complications associated with diabetes.

## Methods

### Cell culture and treatments

Primary human umbilical vein endothelial cells (HUVECs) were obtained from American Type Culture Collection (ATCC; PCS-100-010) and cultured in Vascular Cell Basal Medium (ATCC; PCS-100-030) supplemented with Endothelial Cell Growth Kit (ATCC; PCS-110-041), penicillin (100 U/ml), and streptomycin (100 U/ml) (ATCC; Cat# 30–2300). Cells were maintained at 37 °C and 5% CO2 in a humidified incubator. HUVECs were pre-treated for 2 h with SP6 (100 µg/mL), a dose able to evoke the vascular sub-maximal effect of the peptide [20], and cultured for 48 h with high glucose (30 mM).

### Wound healing assay

HUVECs were seeded at a density of 1 × 10⁵ cells/mL in 60 mm tissue culture dishes and cultured to 80–90% confluence. The cells were then serum-starved for 24 h to synchronize their growth. A uniform wound was created in the confluent monolayer using a sterile 1000 µL pipette tip, positioned perpendicularly to the well surface and dragged in a single, straight direction. After the scratch, the cells were washed twice with PBS to remove detached cells, and then incubated in endothelial basal medium containing 2% FBS. The cells were pretreated with SP6 at 100 µg/ml for 2 h before being exposed to the hyperglycemic environment for 48 h. Cell migration and wound closure were monitored by taking images of the wound area at time 0, 12, 24, and 48 h using a light microscope (EVOS FL; Life technologies).

### Cell transfection

HUVECs were transfected with LPCAT1 siRNA (50 nM) (Santa Cruz Biotechnology, USA; Cat# sc-91777) by using Lipofectamine RNAiMAX (Invitrogen; Thermo Fisher Scientific, USA; Cat# 13778-075) following the manufacturer’s instruction. Cells were kept in a reduced serum medium (Opti-MEM, Life Technologies; Cat# 31985-047) for 16 h of transfection duration followed by a medium change. At the end of the cell transfection HUVECs were pre-treated for 2 h with SP6 (100 µg/mL) and then cultured for 48 h with high glucose (30 mM).

### β-cell culture and glucose-stimulated insulin secretion (GSIS) assay

β-TC-6 cells line was purchased from American Type Culture Collection (ATCC; CRL-3605) and cultured in Dulbecco’s Modified Eagle’s Medium (ATCC; 30–2002) supplemented with 15% FBS (ATCC, 30–2020), penicillin (100 U/ml), and streptomycin (100 U/ml) (ATCC; Cat# 30–2300). Cells were maintained at 37 °C and 5% CO2 in a humidified incubator.

β-TC-6 cells were transfected with LPCAT1 siRNA (100 nM) (Santa Cruz Biotechnology, USA; Cat# sc-149020) using Lipofectamine RNAiMAX (Invitrogen; Thermo Fisher Scientific, USA; Cat# 13778-075) following the manufacturer’s instructions. Cells were kept in a reduced serum medium (Opti-MEM, Life Technologies; Cat# 31985-047) for 16 h of transfection duration, followed by a medium change. β-TC-6 cells were seeded onto a 6-well plate at a density of 5 × 10⁵ cells per well and transfected with LPCAT1 siRNA. Cells were pre-treated with SP6 (100 µg/mL) for 2 h and then exposed to either euglycemic conditions (5.5 mM glucose; Control, siRNA LPCAT1, and SP6 groups) or hyperglycemic conditions (30 mM glucose; high glucose, high glucose + SP6, and high glucose + SP6 + siRNA LPCAT1 groups) for 48 h. After these pre-treatments, the medium was replaced, and cells were starved in fresh medium for 6 h, followed by stimulation with 30 mM glucose for 1 h. The media was collected at 0, 15, 30, and 60 min after glucose stimulation, and used to determine insulin concentration. Secreted insulin levels were measured using a Mouse insulin ELISA kit (Mercodia, Sweden).

### Determination of ATP in β-cells

400 µL of samples were freeze-dried overnight, reconstituted in 100 µL of H2O and vortexed for 30 s. 300 µL of ice-cold MeOH 80% (v/v) containing a mixture of deuterated standards was spiked to the samples, vortexed for 10 min, transferred to an ultrasonic bath for 10 min, and then incubated at −20 °C for 30 min. After centrifugation, 280 µL of supernatants were collected and evaporated using a SpeedVac (Savant, Thermo Scientific, Milan, Italy). Dried extracts were dissolved 100 µL of ACN/H2O 90/10 (v/v), prior to LC-MS analyses.

### Metabolomics profiling

Metabolome analyses were performed on a Thermo Vanquish Flex UHPLC (2.1 mm I.D setup) coupled online to a hybrid quadrupole Orbitrap Exploris 120 mass spectrometer (Thermo Fisher Scientific, Bremen, Germany) equipped with a heated electrospray ionization probe (HESI II). The MS was daily calibrated by Pierce™ FlexMix™ calibration solutions in both polarities. Metabolites separation was performed with an Acquity BEH-Z HILIC (100 × 2.1 mm; 1.7 μm, 100Å) protected with a Acquity BEH-Z HILIC precolumn (5.0 × 2.1 mm; 1.8 μm, 100Å) (Waters, Milford, MA, U.S.A). The column temperature was set at 45 °C, a flow rate of 0.4 mL/min was used, mobile phase consisted of (A): 95:5 H2O/ACN + 10 mM NH4HCOO and (B): ACN/H2O (95:5) (v/v) + 10 mM NH4HCOO were used for both positive and negative ionization mode. The following gradient was employed: 0–0.1 min, 99% B; 0.1–8 min, 99–50% B; 8.0–8.5 min, 50–30% B; 8.5–9.5 min isocratic at 20% B; returning to 99% in 0.1 min, and then 4 min to recondition the column. The ESI source parameters were: sheath gas pressure, 40 a.u; aux gas flow 13 a.u.; sweep gas flow, 0 a.u. Spray voltages were set to 3.3 kV and 3.0 kV for ESI (+) and ESI (-) respectively; the Ion Transfer Tube (ITT) and Vaporizer temperature were set to 280 °C and 300 °C. MS data acquisition was performed in full scan-data dependent acquisition (FS-DDA) in the m/z 70–800, MS1 resolution was set to 60,000, the AGC target was set to auto with a maximum injection time at 100 ms. MS/MS was employed with an isolation window of 1–5 Da, dynamic exclusion of 10 s, resolution was set to 15.000, and HCD was used with normalized collision energies of 20, 40 and 60. The instrument was externally calibrated daily with FlexMix solution (Thermo Fisher) while at the beginning of every LC run the internal calibrant was injected (IC run start mode). FreeStyle (Thermo Fisher Scientific) was used to visualize and perform qualitative analysis on the RAW data, which were then imported to Compound Discoverer v.3.3 (Thermo Fisher Scientific) to normalize, align, detect and identify compounds. Features were extracted from 0 to 14 min chromatography runs, in the m/z = 70–800 mass range. Data were aligned according to an adaptive curve alignment model. Compounds were detected using the following parameters settings: mass tolerance was set to 5 ppm, while retention time tolerance was set to 0.2 min; minimum peak intensity was set to 100,000 AU and the signal-to-noise threshold for compound detection was set to 5. The peak rating filter was set to 3. To perform blank subtraction, we maintained max sample/max blank > 5. To predict elemental compositions of the compounds, the relative intensity tolerance was set to 30% for isotope pattern matching. For the mzCloud database search, both the precursor and fragment mass tolerance were set to 5 ppm. The databases used for matching compounds in ChemSpider for structural search were BioCyc, the Human Metabolome Database and KEGG, and the mass tolerance in ChemSpider Search was set to 5 ppm. The mass tolerance for matching compounds in Metabolika pathways was set to 5 ppm. Compounds were assigned by comparing annotations using the following nodes in order of priority: (1) mzCloud (2) Predicted Compositions; (3) MassList search; (4) ChemSpider Search; (5) Metabolika search. Metabolites intensities were normalized by median, log-transformed and autoscaled for multivariate data analysis.

### Protein extraction and western-blotting

After 48 h of treatment the cells were washed with PBS (ATCC 30–2200) and lysed with M-PER Mammalian Protein Extraction Reagent (Thermo Fisher Scientific, USA; Cat# 78501) containing 1% Halt Protease Inhibitor Cocktail (Thermo Fisher Scientific, USA; Cat# 78429) to obtain the total amount of proteins. The extraction was gently vortexed for 3 min and centrifuged at 14,000 g for 10 min at 4 °C. To isolate cytosolic and membrane proteins, cells were lysed using Mem-PER™ Plus Membrane Protein Extraction Kit (Thermo Fisher Scientific, USA; Cat# 89842), following manufacturer’s instructions. Briefly, the cells were washed with PBS and detached using Trypsin-EDTA (ATCC; PCS-999-003) then centrifuged at 1200 rpm for 3 min. The pellet was washed with Cell Wash and incubated with permeabilizing buffer for 30 min at 4 °C using a shaker, followed by centrifugation at 16,000 g for 15 min at 4 °C to collect the supernatant containing cytosolic proteins. Solubilisation buffer was added to the pellet and mixed by pipetting. The mixture was incubated for one hour at 4 °C and centrifuged at 16,000 g for 15 min at 4 °C to collect the membrane proteins. Protein concentration was evaluated using the Bradford assay (Bio-Rad Laboratories, USA; Cat# 1703932) and multimode microplate readers (Infinite Pro M200, TECAN, Switzerland). Protein samples were separated by 10 or 15% sodium dodecyl sulfate–polyacrylamide gel electrophoresis (SDS-PAGE) and transferred onto a nitrocellulose membrane (Bio-Rad Laboratories, USA; Cat# 1704158). After transferring, membranes were blocked with 5% milk in tris-buffered saline with tween-20 (TBST) for 1 h at room temperature. Blocked membranes were incubated with primary antibodies in TBST and 3% milk at 4 °C overnight. The primary antibody used were anti-API5 (Abcam, UK; Cat# ab65836) diluted at 1:1000; anti-VEGF (Santa Cruz Biotechnology, USA; Cat# sc-7269) diluted at 1:500; anti- LPCAT2 (Proteintech Group, USA; Cat# 15082-1-AP) diluted at 1:5000; anti- LPCAT1 (Proteintech Group, USA; Cat# 16112-1-AP) diluted at 1:1000; anti-GLUT1 (Cohesion Biosciences, UK; Cat# CPA2071) diluted at 1:1000; anti-GLUT4 (ABclonal, DE; Cat# A7637) diluted at 1:1000; Anti-VE Cadherin (Abcam, UK; Cat# ab33168) diluted at 1:500. Primary antibodies anti-GAPDH (ABclonal, DE; Cat# AC027) diluted at 1:5000 and anti- alpha-Tubulin (ABclonal, DE; Cat# AC007) diluted at 1:5000, were used for normalization. The membranes were washed with TBST and incubated with anti-mouse or anti-rabbit IgG (HRP) secondary antibodies (Thermo Fisher Scientific, USA; Cat# 31430, cat# 31460) for 1 h at room temperature. The membranes were washed with TBST and the proteins were visualized using the ChemiDoc™ MP Imaging System (Bio-Rad Laboratories, Inc, CA, USA) with enhanced chemiluminescence substrate (ECL) (Thermo Fisher Scientific, USA; Cat# 34580). Semi-quantitative analysis was performed using Fiji software.

### Immunofluorescence

HUVECs were seeded onto µ-Slide 8 Well (Ibidi, DE; Cat# 80827) subjected to LPCAT1 siRNA transfection, pretreated with SP6 (2 h), and then with high glucose (48 h). Cells were fixed with 4% of paraformaldehyde for 15 min at room temperature. HUVECs were permeabilized with 0,005% saponin in PBS, blocked with 5% BSA for 25 min, and incubated overnight at 4 °C with the following primary antibodies: rabbit polyclonal LPCAT1 antibody (Proteintech Group, USA; Cat# 16112-1-AP), rabbit polyclonal Glucose Transporter Glut1 antibody (Cohesion Biosciences, UK; Cat# CPA2071), mouse monoclonal Ve-Cadherin (Santa Cruz Biotecnologies, USA; Cat# sc-9989). Cells were incubated with secondary antibodies DyLight 549 conjugated anti-rabbit IgG (Vector Laboratories, USA; Cat# DI-1549) and DyLight 488 conjugated anti-mouse IgG (Vector Laboratories, USA; Cat# DI-2488). Images were acquired using a confocal laser-scanning fluorescence microscope TCS SP5 (Leica Microsystems). Nuclei were stained with DAPI.

The colocalization rate was calculated as the percentage of overlap between the fluorescence signals. This was determined by dividing the colocalization area—the region where both fluorescent signals overlap—by the foreground area, which is the total image area minus the background. To ensure accuracy, the colocalization analysis used grayscale confocal images with consistent threshold values applied equally to both channels across all images. The cytofluorograms in Figures illustrate the intensity distribution of fluorescent signals from each channel, shown as individual dots, representing the degree of signal overlap.

### Merocyanine 540

HUVECs were seeded onto a 48-well, subjected to LPCAT1 siRNA transfection, and then pretreated with SP6 (2 h) and high glucose (48 h). Merocyanine 540 (Santa Cruz Biotechnology, USA; Cat# sc-215303) was added to each well at the final concentration of 10 µM and incubated for 10 min at 37 °C and 5% CO2 in a humidified incubator protected from light. An emission scan was performed ranging from 550 to 750 nm with fluorescence excitation set at 540 nm on a Tecan M2000 plate reader.

### Animal model

*Sex as a biological variable*. Our study examined male mice because they exhibit less phenotypic variability. It is unknown whether the findings are relevant for female mice.

All procedures were by the Ethical Committee for Animal Studies of the University of Salerno (Ethical protocol code: 713/2015-PR). C57BL/6J mice were purchased from Charles River Laboratories International, Inc. Four-week-old male C57BL/6J mice were randomized to SD or HFD plus STZ, cages with corn-cob bedding and water and food ad libitum. SP6 pretreatment was performed at 5 mg/Kg daily by gavage, which represents a functional dose that does not affect the blood pressure levels [20]. Cages were housed at 20 ± 2 °C; 12:12 h light: dark cycle and monitored daily by veterinarian staff. At 8 weeks of age, mice were switched to SD or HFD. STZ was administered as described previously [21]. Briefly, mice received a first STZ dose at 13 weeks of age (100 mg/kg body weight, i.p.; Cat# 329420; BD Biosciences, San Jose, CA, USA).

### Isolation of ECs and SMCs from mice arteries

The mesenteric arteries were removed from anaesthetized and heparinized mice and placed in phosphate-buffered saline (PBS, Dulbecco without Ca^2+^ or Mg^2+^). Under a dissecting microscope the vessels were cleaned of connective tissues and periadventitial fats. For mouse mesenteric artery ECs, the vessels were opened longitudinally, cut into two or three small (1- to 2-mm) pieces, and placed, intima side down, on Matrigel-coated 24-well plates (Corning, USA; Cat# 356231). The explants were cultured in Vascular Cell Basal Medium (ATCC; PCS-100-030) supplemented with Endothelial Cell Growth Kit (ATCC; PCS-110-041) and incubated at 37 °C in humified CO₂ until endothelial cells migrated from the vessels. When the cells reached 90% confluence, the medium and the vessels were removed, and the cells were incubated with a dispase solution (0.1 ml/cm2) (Sigma -Aldrich, USA; Cat# D4818, 2 U/ml). Endothelial cells were collected and centrifuged for five minutes at 500 g and transferred to six-well plates (passage 1). Cells were cultured until the end of the experiment.

Mouse primary mesenteric artery SMCs were isolated by incubating the vessels in collagenase II 1 mg/mL (MedChemExpress, USA; Cat# HY-E70005B,) for 30 min at 37 °C. After the removal of the endothelial and adventitia layer, the arteries were further digested with collagenase II for 30 min at 37 °C. The digested tissues were centrifuged at 1800 g for 10 min. Cells were resuspended in DMEM medium (Gibco, Thermo Fisher Scientific, USA; Cat# 41966-029) supplemented with 10% FBS (ATCC; Cat# 30–2025) penicillin (100 U/ml), and streptomycin (100 U/ml) (ATCC; Cat# 30–2300) (passage 1). Cells were cultured until the end of the experiment.

### Glucose tolerance test (GTT) & insulin tolerance test (ITT) assay

Blood samples from fasting mice were collected at time 0 and at 30-minute intervals for 120 min. According to the manufacturer’s protocols, glucose levels were measured using a Mouse Glucose Assay Kit (Crystal Chem, USA; Cat# 81692). Insulin levels were assessed using an Ultra Sensitive Mouse Insulin ELlSA Kit (Crystal Chem, USA; Cat# 90080) following the manufacturer’s instructions. Absorbance was assessed with Tecan Infinite Pro M2000 microplate reader.

To quantify the dynamic change in glycemia independently of baseline differences, and therefore assess exclusively the glucose variation induced by the test, the incremental area under the curve (iAUC) was calculated. For each mouse, the baseline glycemia (time 0) was subtracted from all subsequent time points before integration. This approach was applied to all experimental groups.

### Vascular reactivity studies

Vascular reactivity studies were performed in ex vivo murine mesenteric arteries of wild-type or type 2 diabetic mice as previously reported [22]. Second-order branches of the mesenteric arterial tree were isolated and dissected. Subsequently, the adventitial fat was meticulously removed, and arteries were sectioned into segments. The vessels were then mounted in a pressure myograph system (Danish Myo Technology, Hinnerup, Denmark) containing Krebs solution (95% O₂–5% CO₂, pH ~ 7.40) and maintained at a stable temperature of 37 °C. Vessels were equilibrated by gradually increasing the intraluminal pressure via the P1 valve, adjusting pressure levels sequentially from 5 mmHg to 10, 20, 40, and 60 mmHg at 20-minute intervals. After a 45-minute equilibration period, arterial segments were maintained at 60 mmHg and pre-constricted using phenylephrine (1 × 10⁻⁹ to 10⁻⁵ M) until a stable contraction was achieved. The vessels were then rinsed at least three times to ensure tissue stabilization. Following equilibration, vascular responses were evaluated by inducing pre-constriction with increasing concentrations of phenylephrine (10⁻⁹ to 10⁻⁵ mol/L; Sigma-Aldrich). Endothelium-dependent relaxation was assessed by measuring vasodilation in response to cumulative doses of acetylcholine (ACh) (10⁻⁹ M to 10⁻⁵ M), while endothelium-independent relaxation was determined using nitroglycerin (NG) (10⁻⁹ M to 10⁻⁵ M) in vessels pre-constricted to 80% of their maximal KCl-induced contraction.

### Lipidomics analysis

LC–MS grade water (H_2_O), acetonitrile (ACN), methanol (CH_3_OH), isopropanol (IPA), chloroform (CHCl_3_), methyl tert-butyl ether (MTBE), butanol (BuOH), LC-MS grade additives formic acid (HCOOH) and ammonium formate (HCOONH_4_) were purchased from VWR (Milan, Italy). Quantitative Mass Spec Internal Standard (EquiSPLASH™ and SPLASH™ LIPIDOMIX™) and authentic lipid standard mixtures (LightSPLASH^®^) were purchased by Avanti Polar Lipids (Alabaster, AL, U.S.A). Unless stated otherwise other reagents were all purchased by Merck.

### Lipidome extraction from cells, tissues and plasma

Before extraction, samples from different study groups were randomized. Lipids were extracted from the different matrices as follows: Cells and mouse tissues (pancreas and liver): A volume of 226 µL of ice-cold MeOH containing a mix of deuterated standards was added to either lyophilized cells or 10 mg of lyophilized tissue. Samples underwent two cycles of incubation at −30 °C for 1 min followed by sonication in a sonic bath for 10 min. Subsequently, 750 µL of ice-cold MTBE was added and the samples were incubated in a Thermomixer (Eppendorf) at 4 °C and 550 rpm for 1 h. Afterward, 188 µL of H_2_O was added and the samples were centrifuged to separate the upper phases, which were dried using a SpeedVac (Savant, Thermo Scientific, Milan, Italy). Lipid extracts from cells, pancreas and liver were reconstituted in 100, 230, and 280 µL of CHCl_3_/MeOH/IPA (1/2/4, v/v), respectively. Results were expressed as pmol/µg of protein.

Plasma: Briefly, 20 µL of plasma were thawed on ice and mixed with 225 µL of ice-cold MeOH containing a mixture of deuterated standards, followed by vortexing for 10 s. Next, 750 µL of cold MTBE were added and the samples were agitated in a Thermomixer (Eppendorf, Milan, Italy) for 10 min at 300 rpm and 4 °C. Subsequently, 188 µL of H_2_O were added and the mixture was vortexed for 20 s before being centrifuged at 14,680 rpm for 10 min at 4 °C to achieve phase separation. The upper layer was carefully collected and evaporated using a SpeedVac (Savant, Thermo Scientific, Milan, Italy). The dried extracts were reconstituted in 100 µL of BuOH/IPA/H_2_O (8/23/69, v/v %) and analyzed using UHPLC-TIMS-MS. Results were reported as µM.

### Untargeted lipidomics

UHPLC-TIMS-MS analyses were performed on an Ultimate RS 3000 UHPLC (Thermo Fisher Scientific, Milan, Italy), which comprised a RS 3000 autosampler, column oven and binary pump with a 35 µL mixer. All connections were Viper (Thermo Fisher Scientific, USA) stainless steel capillaries (0.100 mm I.D.). The column outlet was connected to the MS source with a peek tubing (0.100 mm I.D.) of the shortest length possible. The UHPLC system was coupled online to a TimsTOF Pro Quadrupole Time of Flight (Q-TOF) (Bruker Daltonics, Bremen, DE) equipped with an Apollo II electrospray ionization (ESI) probe. The separation was performed with an Acquity UPLC CSH™ C18 column (50 × 2.1 mm; 1.7 μm, 130 Å) protected with a VanGuard CSH™ precolumn (5.0 × 2.1 mm; 1.7 μm, 130 Å) (Waters, Milford, MA, U.S.A). The column temperature was set at 65 °C, a flow rate of 0.55 mL/min was used, mobile phase consisted of (A): ACN/H_2_O 60:40 (v/v %) and (B): IPA/ACN 90:10 (v/v %) both buffered with 10 mM HCOONH_4_ and 0.1% HCOOH. The following gradient has been used: 0 min, 40% B; 0.4 min, 43% B; 0.425 min, 50% B; 0.9 min, 57% B; 2.0 min, 70% B; 2.950 min, 99% B; 3.3 min, 99% B; 3.301, 40% B and then 0.7 min for column re-equilibration. The TIMS-MS analyses were performed in data-dependent parallel accumulation serial fragmentation (DDA-PASEF) with positive ionization and each sample was injected in triplicate. The injection volume was set at 2 µL. Mass spectra were recorded in the range m/z 100–1500, with an accumulation and ramp time to 100 ms each. The ion mobility was scanned from 0.55 to 1.80 Vs/cm^2^. Exclusion time was set to 0.1 min, Ion charge control (ICC) was set to 7.5 Mio. The total acquisition cycle was of 0.32 s and comprised one full TIMS-MS scan and two PASEF MS/MS scans MS/MS spectra were acquired with a TIMS-MS-STEPPING ion mobility-dependent collision energy mode: CE [eV] #1: 20–40 and CE [eV] #2: 35–50. Source parameters: Nebulizer gas (N_2_) pressure: 4.0 Bar, Dry gas (N_2_): 10 L/min, Dry temperature: 220 °C.

### Lipidomics data analysis and processing

4D data alignment, filtering and annotation were performed with MetaboScape 2023b (Bruker) employing a feature finding algorithm (T-Rex 4D) that automatically extracts buckets from raw files. A mixture (1:1 v/v) of 10 mM sodium formate calibrant solution and ESI-L Low Concentration Tuning Mix was injected before each run to recalibrate the mass and mobility data, respectively. The feature detection threshold was set to 250 counts, and the minimum number of data points in the 4D-TIMS space was set to 100. In addition, a recursive feature extraction was used (75 points).

Lipid annotation was performed first with a rule-based annotation, based on characteristic fragments and their intensity in acquired MS/MS spectra, and, subsequently, using the LipidBlast spectral library of MS DIAL (http://prime.psc.riken.jp/compms/msdial/main.html) with the following parameters: Mass accuracy: narrow 2 ppm, wide 10 ppm; mSigma: narrow 30, wide 250, MS/MS score: narrow 800, wide 150. Collision cross-section (CCS)%: narrow 2, wide 3.5. The spectra were processed in positive mode using [M + H]^+^, [M + Na]^+^, [M + K]^+^, [M + H–H_2_O]^+^ and [M + NH_4_] + ions. CCS values were compared with those predicted by CCSbase platform (https://ccsbase.net/), the assignment of the molecular formula was performed for the detected features using Smart Formula™ (SF). Each lipid feature was manually curated following Lipidomics Standard Initiative (LSI) guidelines (https://lipidomics-standards-initiative.org/guidelines/lipid-species-identification/general-rules). LipidCreator tool (https://lifs-tools.org/lipidcreator.html) extension in Skyline (https://skyline.ms/project/home/begin.view) was used for in silico comparison of specific product ions for manual MS/MS curation. Supplementary Tables 1–4 report lipid annotation in different samples.

### MALDI-MSI analysis

#### Cryosectioning

Mouse pancreas tissue sections, 12 μm thick, were cut at −18 °C upon one-hour conditioning using a cryostat microtome (Leica CM3050S, Leica Microsystems, Wetzlar, DE) and subsequently thaw-mounted onto pre-cooled indium tin oxide (ITO) coated glass slides (Bruker Daltonics, Bremen, DE) and stored at −80 °C until further use. Sections were desiccated at room temperature 1 h prior to acquiring the optical images on a reflecta^®^ MF5000 scanner (reflecta^®^, DE) with HistoView Tissue Scanner II software v1.00.90.

#### Sample Preparation for MALDI-MSI analysis of insulin and ATP

For MALDI-MSI of insulin, a 6 mL solution of 35 mg/mL 2,5-Dihydroxybenzoic acid (DHB) in 50% ACN plus 0.2% TFA was homogeneously applied over the tissues using an automated sprayer (TM sprayer, HTX Technologies) in six passes, at 95 °C, with a nitrogen gas pressure of 10 psi, flow rate of 70 µL/min, nozzle velocity of 1100 mm/min and 2 mm track spacing. For MALDI-MSI analysis of polar metabolites, the 9-aminoacridine (9-AA) matrix was dissolved in 70% EtOH in H 2 O (concentration 10 mg/mL). An automated pneumatic sprayer (TM-Sprayer, HTX Technologies, Chapel Hill, NC, USA) was used to spray the solution over the tissue sections in four passes at 90 °C, with a nitrogen gas pressure of 6 psi, a flow rate of 120 µL/min, a nozzle velocity of 1200 mm/min, 2 mm of track spacing, and a CC spray pattern.

#### MALDI-MSI analysis and data processing

MSI analyses were carried out with a rapifleX MALDI Tissuetyper TOF/TOF MS system (Bruker Daltonics, Bremen, DE) equipped with a Smartbeam 3D laser under “Single”. 1000 laser shots position in 50 μm spatial resolution experiments were collected per spot. The laser power was optimized at the start of each acquisition with a repetition rate of 5 kHz. The analyzer was operated in reflector mode. The measurements were performed in a positive mode across the m/z range of 2000–6000 with a digitizer frequency of 1.25 GHz. Tissue sections were analyzed in a random order to prevent any possible bias due to matrix degradation or variation in mass spectrometer sensitivity. The method was externally calibrated using a using red phosphorus dissolved in 50% acetone and spotted beside the tissue sections.

The insulin signal was identified at m/z 5805 and the anatomical distribution was used for confirmation. All MSI data were visualized using flexImaging (Bruker Daltonics, version 6.0). TOF spectra were normalized to the total ion count (TIC) of each individual spectrum. The reduced spectra were imported into SCiLS lab software (Version 2021a, GmbH, Bremen, DE) and the mean ion intensity for each compound in the average mass spectra of the region of interest (either the whole tissue section or a specific brain region) was exported and used for data exploration and statistical analysis. ATP MSI analysis was performed as reported elsewhere [23].

#### Hematoxylin and Eosin (H&E) stains to MSI Co-Registration

Following MSI analysis, the MALDI matrix was removed by submerging the slide in 95% ethanol for 30 s, then the sections were fixed in two successive 5 min washes in 100% ethanol and were stained by H&E. The slides were cover slipped and scanned using a reflecta^®^ MF5000 scanner (reflecta^®^, DE). The whole slide H&E images were imported to SCiLS and overlaid with the MSI data.

#### Synthesis of SP6-FAM

The synthesis of SP6-FAM peptide was performed with a solid phase approach using a standard Fmoc methodology on a Biotage Initiator + Alstra automated microwave synthesizer (Biotage, Uppsala, Sweden). SP6-FAM was synthesized on an Fmoc-L-Ile-Wang resin (0.6–0.7 mmol/g, 100 mg), previously deprotected with 30% piperidine/DMF (1 × 3 min, 1 × 10 min) at room temperature (rt). The resin was then washed with DMF (4 × 4.5 ml) and protected amino acids added on to the resin stepwise. Coupling reactions were performed using Nα-Fmoc amino acids (4.0 eq., 0.5 M), HBTU (3 eq, 0.6 M), HOAt (3 eq, 0.5 M), and DIEA (6 eq, 2 M) in N-methyl-2-pyrrolidone (NMP) for 10 min at 75 °C (2×). After each coupling step, the Fmoc protecting group was removed as described above. The resin was washed with DMF (4 × 4.5 ml) after each coupling and deprotection step. Finally, the N-terminal Fmoc group was removed, resin-bound peptide was coupled with 5(6)-carboxyfluorescein (FAM) using DIC (6 eq) and HOB*t* (3 eq) for 20 min at 60 °C. The resin was washed with DCM (7x), and the peptide released from the resin with TFA/TIS/H_2_O (ratio 95:2.5:2.5) for 2 h. The resin was removed by filtration and the crude peptide recovered by precipitation with cold anhydrous ethyl ether to give a yellow powder that was then lyophilized.

#### Statistical analysis

Data are presented as mean ± S.D. Differences were analyzed using one-way or two-way ANOVA followed by Tukey’s post hoc multiple comparisons test as appropriate for experiments involving ≥ 3 groups. In the in vitro analyses, the levels of LPCs and PCs, measured in pmol/µg of protein, were reported as fold changes relative to the control. In vitro differences in lysolecithin levels were analyzed using one-way ANOVA followed by an uncorrected Fisher’s LSD test to detect even small differences between groups. Differences in vascular reactivity studies, glucose levels, and body weight during treatment were analyzed using two-way ANOVA followed by Tukey’s post hoc multiple comparisons test. A *p*-value of < 0.05 was considered statistically significant. All experiments included at least three biological replicates. Statistical analyses were performed using GraphPad Prism software version 10. No statistical analysis was used to predetermine sample sizes; estimates were based on our previous experience, experimental methodologies, availability, and feasibility to achieve statistically significant results. Experimental mice were randomly assigned to experimental or control groups. Investigators were blinded to the treatment of individual animals during the experiments and in the outcome assessments. Statistical analysis for iAUC was performed using one-way ANOVA followed by Tukey’s HSD post-hoc test for multiple comparisons across groups (Control, Diabetes, SP6 + Diabetes). The incremental AUC (iAUC) was computed in R (https://www.r-project.org/) using the MESS package. Statistical significance was set at *p* < 0.05.

## Results

### SP6 prevents hyperglycemia-induced wound healing impairment by modulating VEGF/API5 signaling

**Fig. 1:**
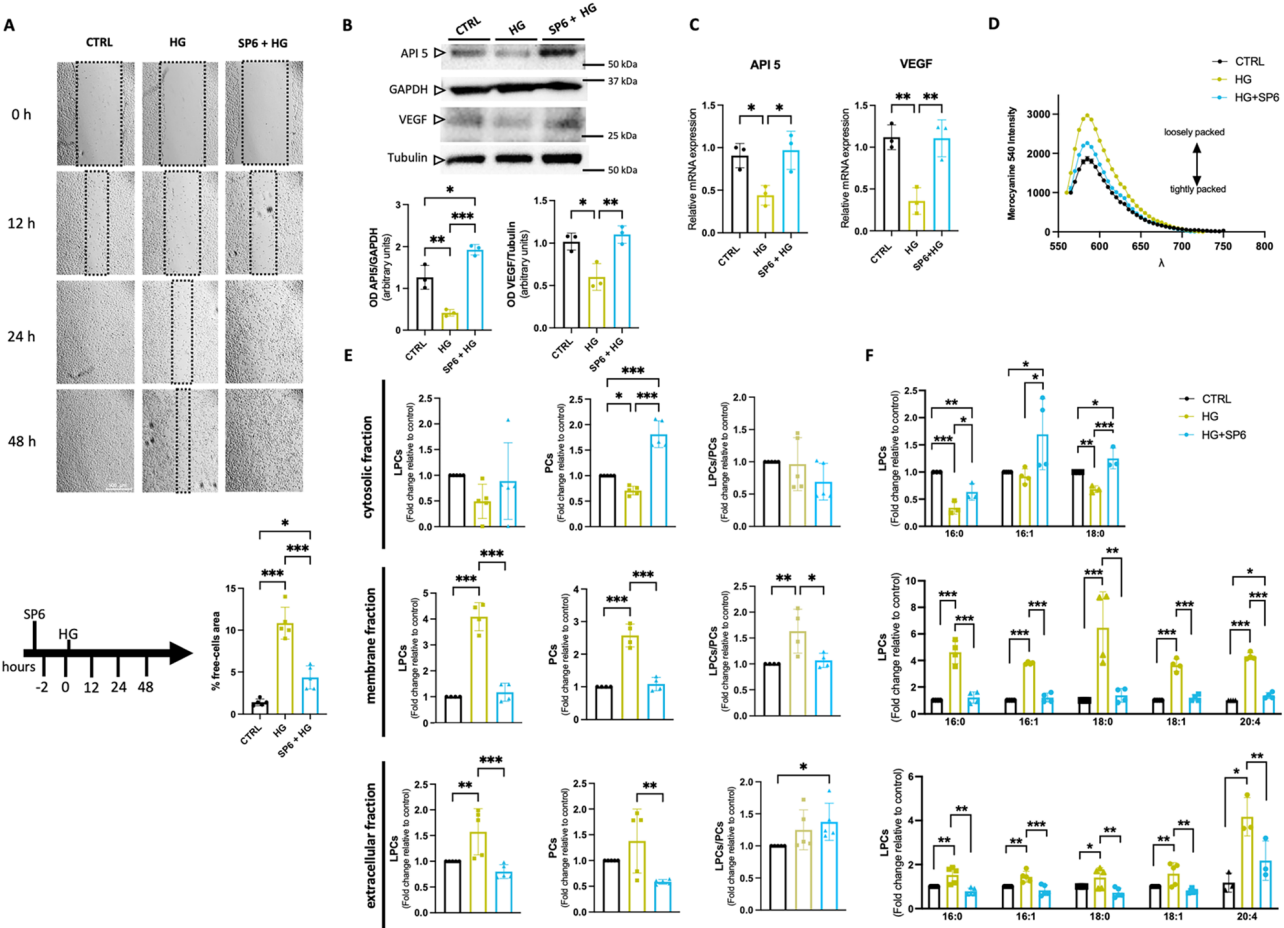
High glucose impairs endothelial function and wound healing by altering VEGF/API5 signaling and lysolecithin homeostasis in subcellular compartments. **A** Representative examples of endothelial cell wound healing experiment in which confluent HUVECs were wounded with a pipette tip and photographed immediately after the wounding (time 0) and after 12 h, 24 h and 48 h after exposure to either vehicle (ctrl), high glucose (HG 30 mM) or SP6 plus HG (100 µg/ml, HG, 30mM). Scale bar: 500 μm. Rectangles indicate margins of wounds. *Bottom*: The arrow indicates the experimental setting; the bar graph reports the % of free-cell area evaluated at 48 h post wounded. **B** Representative immunoblots and densitometric analyses of 3 independent experiments evaluating protein levels of API5 or VEGF normalized on GAPDH or tubulin, respectively. **(C)** Relative mRNA expression is given as relative quantification (RQ = 2^−ΔΔCT^) values of API5 and VEGF. 2^−ΔΔCT^ values of the first controls were normalized to 1. **D** Membrane phospholipid packaging status evaluation by MC540 fluorescence assay in HUVECs. **E**–**F** LC-MS/MS analysis, quantitative profile of LPCs, PCs, total LPC/PC in subcellular fractions, and **F** the most relevant LPCs in cytosolic, membrane, and extracellular fractions of HUVECs, expressed as fold change relative to the control

Using the HUVECs model [24], we observed that a hyperglycemic environment causes endothelial dysfunction related to impaired wound healing after 48 h (Fig. 1A). In contrast, pretreatment with SP6 at 100 µg/mL for 2 h before the exposure for 48 h to hyperglycemia completely abolished the harmful effect of the hyperglycemic environment, restoring the physiological wound healing process (Fig. 1A). We therefore focused on vascular endothelial growth factor (VEGF) and Apoptosis inhibitor 5 (API5), both involved in cell survival in diabetes. Our analysis reveals that, while high-glucose conditions led to downregulation of VEGF at both mRNA and protein levels, SP6 treatment prevented these effects (Fig. 1B–C). Concomitantly, hyperglycemia induced a marked downregulation of API5, a direct intracellular apoptosis regulator, while SP6 pre-treatment preserved its expression in endothelial cells at both protein and mRNA levels (Fig. 1B–C).

### SP6 preserves lysolecithin homeostasis in subcellular compartments of endothelial cells exposed to high glucose levels

The balance between LPCs, PCs, and phospholipids is essential for maintaining cell membrane integrity and function, influencing the apoptotic process in hyperglycemic conditions [25, 26]. The merocyanine assay, a technique to evaluate membrane phospholipid packaging status, revealed that hyperglycemic conditions reduced membrane packaging (Fig. 1D), a phenomenon strictly associated with cellular death [27]. Conversely, SP6 pretreatment shifts membrane packing toward a more tightly packed state, supporting cell-cell communication and promoting the overall health of the cell population (Fig. 1D).

Lipidomic analysis of both cytosolic, membrane, and extracellular fractions of endothelial cells revealed an alteration in the LPCs/PCs ratio in hyperglycemic conditions (Fig. 1E). In particular, the cytosolic fraction showed a downregulation of both LPCs and PCs in hyperglycemic conditions compared to an euglycemic environment (Fig. 1E, *left*). In cells pretreated with SP6 before exposure to high glucose levels, we observed a marked regulation of LPCs and PCs toward increased levels rather than decreased levels, as observed in hyperglycemic conditions (Fig. 1E, *left*). In contrast, in the membrane fraction, during hyperglycemic conditions, we detected a significant increase in LPCs levels, particularly of LPC 16:0, 16:1, 18:0, 18:1 and 20:4 (Fig. 1F, *right*). Notably, this phenomenon was prevented in SP6-pretreated cells, which showed the same levels of LPCs and PCs as control cells (Fig. 1E–F). In the extracellular fraction, we did not detect a modification of lysolecithins between hyperglycemic and SP6 pretreatment conditions, which showed a marked reduction compared to the control condition (Fig. 1E–F).

### SP6 selectively preserves LPCAT1 expression and endothelial integrity

**Fig. 2:**
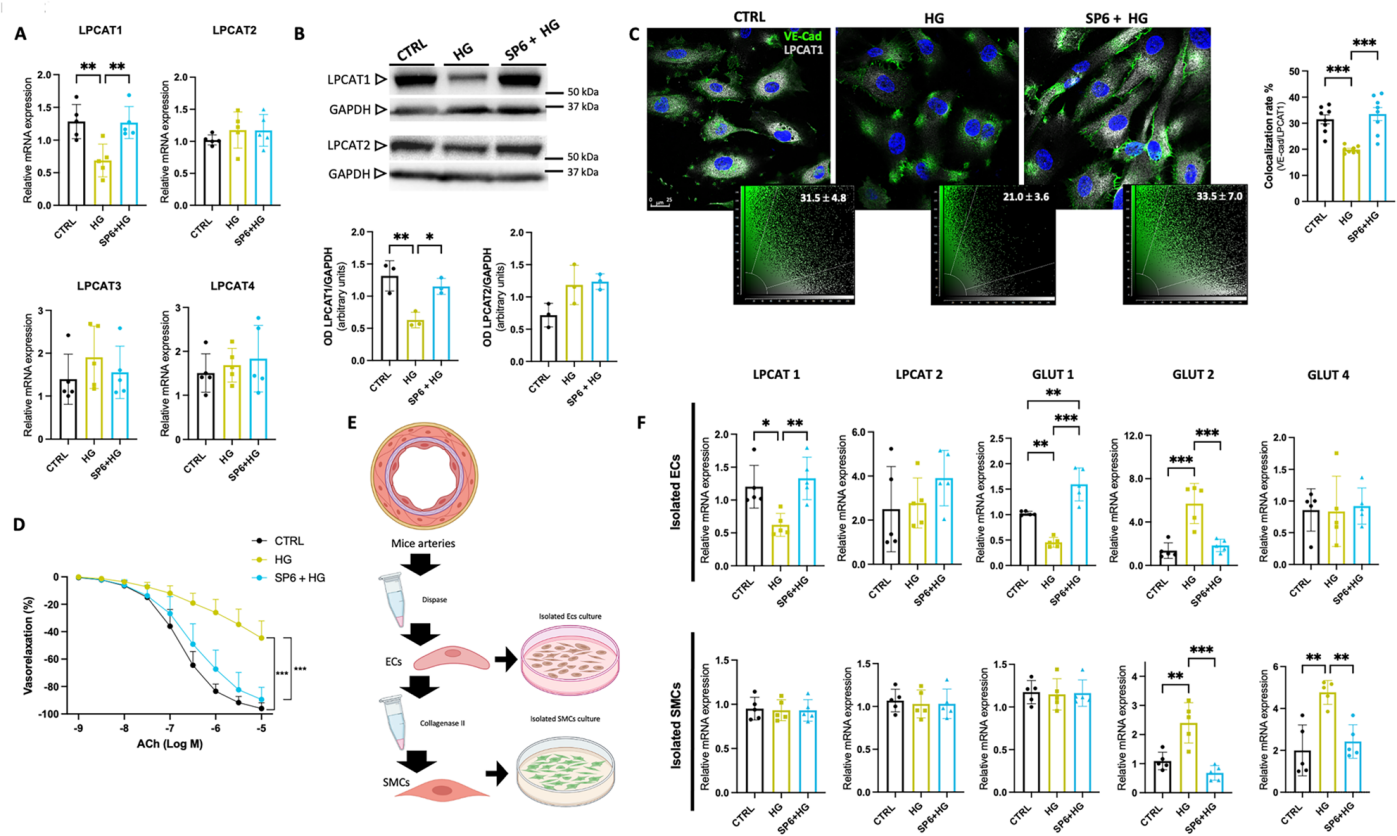
LPCAT1 is involved in hyperglycemia-induced vascular damage and lysolecithin homeostasis.** A** Relative mRNA expression is given as relative quantification (RQ = 2^−ΔΔCT^) values of LPCAT1, LPCAT2, LPCAT3, and LPCAT4. 2^−ΔΔCT^ values of the first controls were normalized to 1. **B** Representative immunoblots and densitometric analyses of 3 independent experiments evaluating protein levels of LPCAT1 and LPCAT2. GAPDH has been used as internal loading control for normalization expression in HUVECs. **C** Immunofluorescence analysis of VE-cadherin and LPCAT1 signaling end relative co-localization plots in HUVECs. Scale bar: 25 μm. **D** Vascular reactivity assay, dose-response curves to acetylcholine (ACh) in ex vivo C57BL/6 mouse mesenteric arteries under basal condition (CTRL) after three hours of high glucose exposure (30 mM) and pretreated with SP6 for two hours before HG exposure. **E** Graphical representation of endothelial cells (ECs) and smooth muscle cells (SMCs) isolation from pooled mice mesenteric arteries exposed to different experimental conditions; **F**: Relative mRNA expression is given as relative quantification (RQ = 2^−ΔΔCT^) values of LPCAT1, LPCAT2, GLUT1, GLUT2, and GLUT4 into isolated ECs (*upper*) and in SMCs (*bottom*). 2^−ΔΔCT^ values of the first controls were normalized to 1

Our data revealed that under hyperglycemic conditions, SP6 can regulate LPCs/PC signaling. We, therefore, investigated the possible modulation of the expression of lysophosphatidylcholine acyltransferase (LPCAT) enzyme isoforms evoked by the peptide. Four isoforms of LPCATs, LPCAT1-4, have been identified in vascular tissues [28]. mRNA analysis on endothelial cells showed that while LPCAT1 mRNA levels decreased in hyperglycemia, SP6 pretreatment prevented this reduction (Fig. 2A). In contrast, no changes in mRNA levels of LPCAT2, LPCAT3, and LPCAT4 were observed (Fig. 2A). Interestingly, protein expression analysis confirmed the downregulation of LPCAT1 under high glucose conditions. This effect was completely prevented by pretreatment with SP6. Conversely, neither hyperglycemia nor SP6 influenced the protein levels of LPCAT2 (Fig. 2B and Supplementary Fig. 1 A). Similarly, protein levels of LPCAT3 did not show any modification, while LPCAT4 resulted in not being detectable in ECs (Supplementary Fig. 1 A), indicating that these latter enzymes are only present at the transcriptomic level in endothelial cells.

Vascular permeability and the integrity of the dynamic architecture of endothelial cells are closely intertwined with the modulation of VE-cadherin (VE-Cad) [29]. Immunofluorescent analyses revealed that the typical membrane distribution of VE-Cad was completely disrupted during high-glucose treatment. In contrast, cells pretreated with SP6 maintain the physiological distribution of VE-Cad, consistent with its key role in the maintenance of endothelial plasticity and membrane integrity (Fig. 2C). Coherently with mRNA and protein expression, in high glucose conditions, we detected a low signal of LPCAT1 and a consequent reduction of VE-Cad/LPCAT1 co-localization rate, thus supporting the critical impact of hyperglycemia on enzyme regulation and the capability of SP6 to prevent its downregulation and cellular localization (Fig. 2C).

### LPCAT1 is essential for endothelial protection mediated by SP6 in ex vivo mesenteric arteries under hyperglycemia

The data obtained so far prompted us to investigate ex vivo the effect of SP6 on endothelial function in mice’s mesenteric arteries. As expected, under hyperglycemic conditions, the endothelial response to acetylcholine showed a significant impairment of vasorelaxation (Fig. 2D). Notably, a two-hour pre-treatment with SP6 effectively reduced endothelial damage caused by three hours of high glucose exposure (Fig. 2D).

To understand the mechanism behind the vascular protection triggered by SP6, we examined its effects on isolated endothelial and smooth muscle cells from ex vivo mesenteric arteries (Fig. 2E). Immunofluorescence for CD105 and alpha-smooth muscle actin confirmed the specificity of the isolated cells (Supplementary Fig. S2). Our results showed that under hyperglycemic conditions, a reduction in LPCAT1 was only detected in endothelial cells, while no significant changes occurred in SMCs (Fig. 2F). In both EC and SMC cells, LPCAT2 mRNA expression levels remained unchanged (Fig. 2F). This supports the involvement of LPCAT1 in SP6’s protective action on the endothelium, consistent with the observed regulation of phosphatidylcholine metabolism. Additionally, assessing the main GLUTs involved in vascular cells indicated that SP6 can enhance the mRNA levels of GLUT1 and preserve GLUT2 levels, without affecting GLUT4 expression in endothelial cells (Fig. 2F).

In contrast, in SMCs, we observed a preservation in both GLUT2 and GLUT4 mRNA levels, whereas LPCAT1 and GLUT1 mRNA expression remained unchanged (Fig. 2E).

Molecular underpinnings in endothelial cells revealed that while high glucose markedly decreased the phosphorylation of AKT^S473^ and eNOS^S1177^. SP6 pretreatment fully preserved their phosphorylation levels. LPCAT1 silencing completely abolished these effects, confirming that SP6 protection requires LPCAT1 integrity (Supplementary Fig. S1A).

### LPCAT1 Silencing abolishes SP6’s protective effects on endothelial membrane integrity and wound healing

**Fig. 3:**
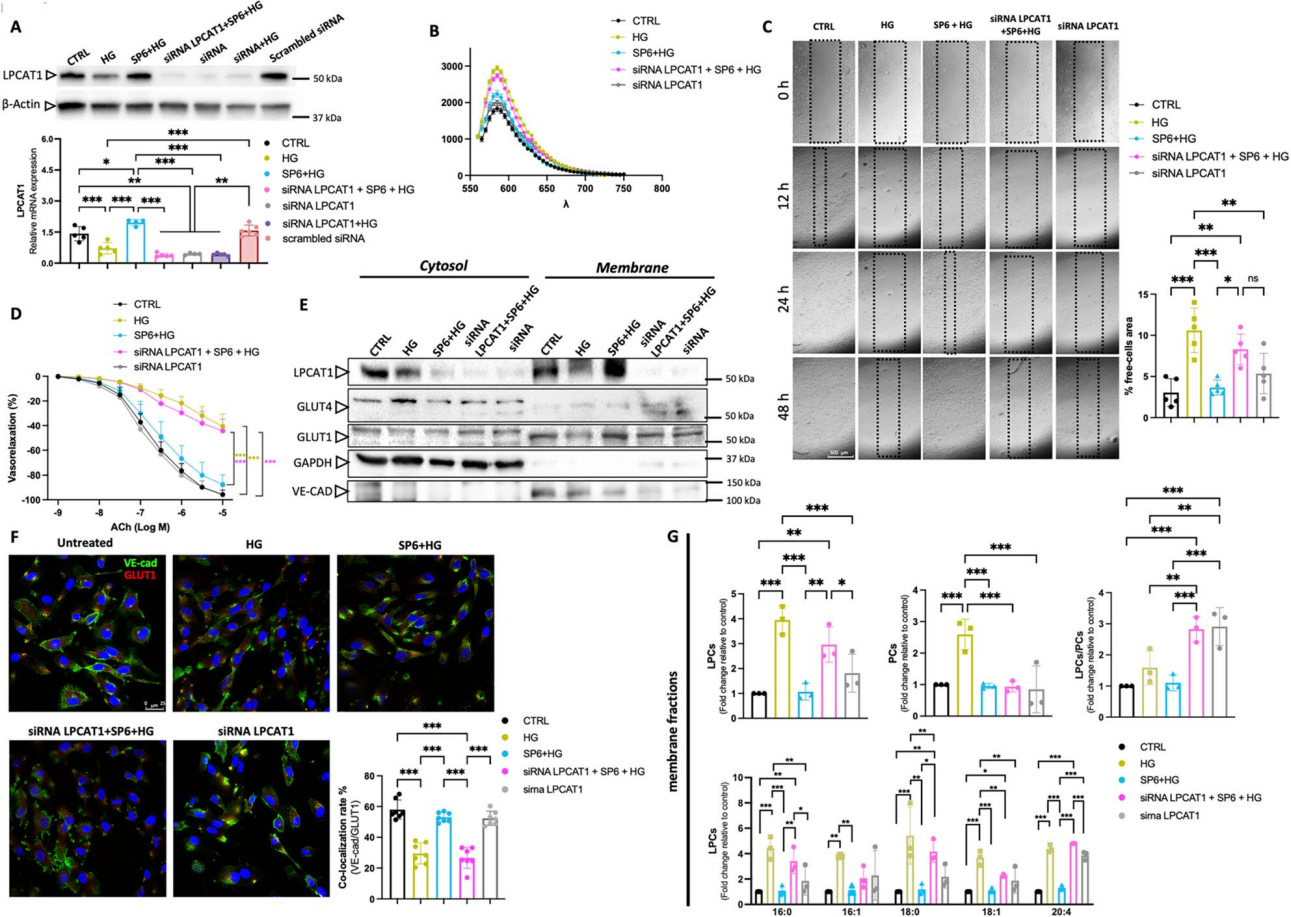
LPCAT1 is a regulator of endothelial function and membrane lipid composition under hyperglycemic conditions. **A** *upper*: Representative immunoblots analyses of the silencing efficacy siRNA of LPCAT1. b-Actin has been used as an internal loading control; *bottom*: Relative mRNA expression is given as relative quantification (RQ = 2^−ΔΔCT^) values of LPCAT1. 2^−ΔΔCT^ values of the first controls were normalized to 1. **B** Membrane phospholipid packaging status evaluation by MC540 fluorescence assay in ECs. **C** Representative images of endothelial wound healing experiments acquired immediately after the wounding (time 0) and after 12 h, 24 h and 48 h after treatment. Rectangles indicate margins of wounds. Scale bar: 100 μm. The bar graph reports the % of free-cell area evaluated at 48 h post wounded. **D** Vascular reactivity assay, dose-response curves to acetylcholine (ACh) in ex vivo C57BL/6 mouse mesenteric arteries in different experimental conditions. **E** Immunoblot analyses in cytosol and membrane fractions obtained from isolated ECs in different experimental conditions. GAPDH and VE-cadherin were used as markers for the separation between the cytosolic and membrane fractions. **F** Immunofluorescence analysis of VE-cadherin and GLUT1 in isolated ECs in different experimental conditions. Scale bar: 25 μm. The bar graph reports the relative co-localization rate (%) of VE-cadherin and GLUT1. **G** LC-MS/MS analysis, quantitative profile of LPC, PC, total LPC/PC, and the most relevant LPCs in membrane fractions of isolated ECs, expressed as fold change relative to the control

Silencing LPCAT1 in endothelial cells resulted in a loss of protein expression, as confirmed by western blot and RT-PCR (Fig. 3A). Additionally, our data indicate that SP6 pretreatment can mitigate high-glucose-induced downregulation of LPCAT1, strengthening the potential link between LPCAT1 and the protective effects of SP6. This notion was further supported by the observation that under high glucose conditions, SP6 was unable to prevent membrane dysregulation in LPCAT1-silenced cells, as demonstrated by merocyanine assay (Fig. 3B). Additionally, in the presence of both high glucose and LPCAT1 siRNA, SP6 failed to promote cell migration and wound closure (Fig. 3C). However, it preserves cell viability (Supplementary Fig. S1B). These findings underscore the critical role of LPCAT1 in endothelial cells and advance it as a key mediator of the beneficial effects of SP6 on endothelial cell function and vascular health.

### SP6/LPCAT1/GLUT1 axis protects against hyperglycemia-induced endothelial dysfunction

Translating these data into an ex vivo approach on mice mesenteric arteries exposed to high glucose levels, we revealed that SP6 pretreatment was able to promote a complete preservation of endothelial vasorelaxation, which is impaired under high glucose conditions. However, this beneficial effect was lost by siRNA-mediated knockdown of LPCAT1 (Fig. 3D).

To clarify how SP6 protects endothelial cells from high-glucose-induced damage, we next investigated the regulation of membrane glucose transporter expression in isolated endothelial cells from mouse mesenteric arteries. Our data confirmed the downregulation of LPCAT1 protein expression in the membrane fraction of mesenteric arteries- EC-derived exposed to high glucose conditions (Fig. 3E). This effect was mitigated by pretreatment with SP6, which preserved LPCAT1 membrane localization and expression. Conversely, silencing LPCAT1 under high-glucose conditions blocks SP6-evoked beneficial effects, leading to a loss of LPCAT1 and GLUT1 membrane localization.

### SP6 preserves GLUT1 membrane localization and lipid composition through LPCAT1-dependent mechanisms

Immunofluorescence analysis confirmed that SP6 pretreatment maintains the proper membrane localization of GLUT1 and VE-cadherin in endothelial cells exposed to high glucose (Fig. 3F). Silencing LPCAT1 further reduced GLUT1/VE-Cad colocalization (Fig. 3F), indicating that LPCAT1 plays a key role in maintaining endothelial barrier function and glucose uptake under high glucose conditions.

Interestingly, analysis of membrane lipid composition revealed that during LPCAT1 silencing, SP6 failed to prevent the increase in LPC levels (16:0, 18:0, and 20:4) observed under hyperglycemic conditions (Fig. 3G), confirming the key role of this enzyme in the protective action of SP6.

### Glucose tolerance and endothelial function are preserved by SP6 pretreatment in a high-fat diet/streptozotocin murine model of type 2 diabetes

**Fig. 4:**
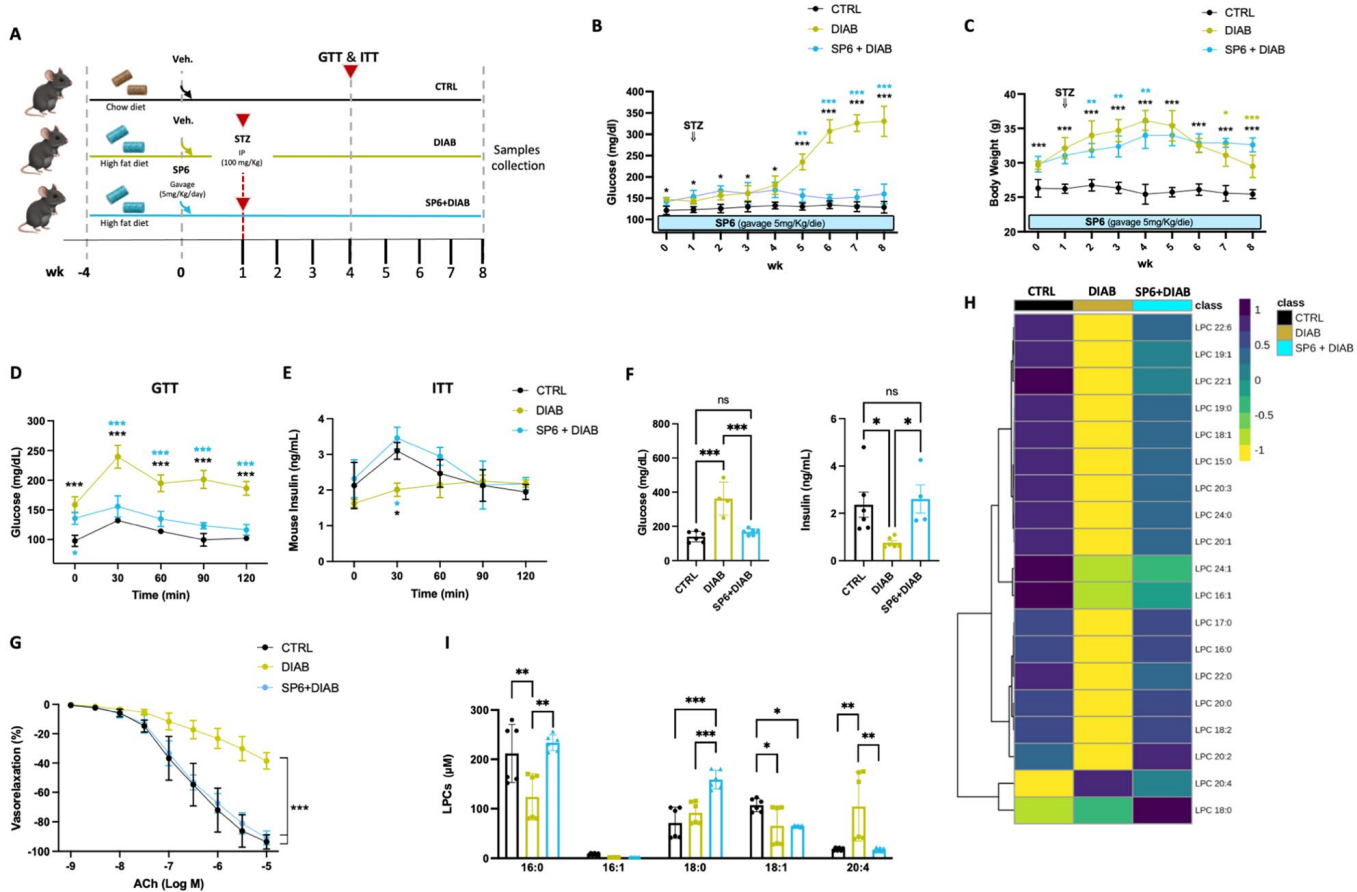
SP6 pretreatment protects against hyperglycemia-induced endothelial dysfunction and lysolecithin dysmetabolism in diabetic mice. **A** Schematic representation of in vivo treatment and time points analyses. **B–C** Weekly fasting blood glucose levels and body weight measurement. **D** Glucose tolerance test (GTT) and **E** insulin tolerance test (ITT) performed at the fourth week of treatment. Data are presented as mean ± SEM. **F** Plasma glucose and insulin concentrations were measured at the end of the 8-week treatment period. **G** Vascular reactivity assay, dose-response curves to acetylcholine (ACh) in ex vivo C57BL/6 mouse mesenteric arteries collected at the end of the 8-week treatment period. **H** LC-MS/MS analysis. Heatmap highlighting the quantitative differential levels of LPC and PC in mice plasma. **I** Quantitative profile of plasmatic most relevant LPCs. All measurements were performed in vehicle-treated mice (CTRL), diabetic mice (DIAB), and SP6-pretreated diabetic mice (SP6 + DIAB). Values are presented as mean ± SEM

To evaluate the effects of the peptide in vivo, we administered SP6 to mice fed a high-fat diet for eight weeks (Fig. 4A), starting one week before a single injection of streptozotocin to induce a model of type 2 diabetes [30–32] Weekly monitoring of glucose levels and body weight showed a slight, expected increase in blood glucose in both the vehicle and SP6-treated groups until week four (Fig. 4B). Surprisingly, in week five, while the glucose levels in the vehicle-treated diabetic group continued to rise until week eight, the SP6-treated diabetic mice displayed blood glucose levels similar to those of the control animals (Fig. 4B). Both diabetic groups initially gained weight, then experienced a significant weight loss starting around week five. This loss was more pronounced in diabetic animals treated with vehicle compared to those treated with SP6 (Fig. 4C).

To elucidate the underlying mechanisms of the aforementioned findings, we performed glucose tolerance tests (GTT) and insulin tolerance tests (ITT) at week four. Diabetic animals treated with vehicle displayed a marked increase in blood glucose levels that persisted for 120 min, similar to the pattern in human patients with impaired glucose tolerance. In contrast, both control and SP6-treated mice experienced a transient hyperglycemia, followed by a rapid return to basal glucose levels (Fig. 4D). Consistently, the insulinemic dosage revealed that insulin levels peaked at 30 min post-injection in both control and SP6-pretreated mice, while they remained significantly lower in diabetic mice (Fig. 4E). Assessment of iAUC, based on blood samples collected at 4th week, show a statistically significant difference in the GTT between the SP6 + DIAB group versus the DIAB group, confirming the positive effect of SP6 on glucose regulation. Although a trend toward reduction was observed in the ITT iAUC in the SP6 + DIAB group, thus suggesting potential impact on insulin tolerance, no statistically significant differences were noted at this time point (Supplementary Fig. S3). Finally, the glycemic and insulinemic dosage performed at the end of the 8 weeks of treatment in the plasma of mice confirmed the preserved insulin secretion and improved glycemic control evoked by SP6 treatment in diabetic mice (Fig. 4F). In addition, endothelial function assessment in mesenteric arteries revealed complete preservation in SP6-treated mice, contrasting with the impaired endothelial vasorelaxation observed in the diabetic group (Fig. 4G).

### Improved plasma lysolecithin dysmetabolism by SP6 pretreatment

Considering the metabolic protection covered by SP6 pretreatment on diabetic induction, we performed lipid profiling analysis in the plasma of mice collected at the end of treatment (Fig. 4H). Interestingly, while the overall levels of circulating LPCs exhibited a decreasing trend in our diabetic model, with both saturated and polyunsaturated species, the level of LPC 20:4, carrying pro-inflammatory arachidonic acid, was significantly elevated in the diabetic group. Notably, treatment with SP6 successfully reversed this increase (Fig. 4I), indeed the Log_2_ fold-change ratios indicate a substantial recovery of circulating LPC levels toward control levels (Supplementary Fig. S4). This trend is observed across different LPC species, considering both the carbon number (y-axis, e.g., LPC 15:0 to LPC 24:0) and the degree of unsaturation (x-axis, e.g., LPC 20:0 to LPC 20:4). This observation prompted us to investigate further lipidomics changes in the pancreas and liver, which represent key metabolic tissues for glycemic control and are crucial in diabetes pathophysiology.

### SP6 counteracts lysolecithin dysmetabolism and preserves pancreatic ATP production and insulin secretion via LPCAT1-dependent pathways

**Fig. 5:**
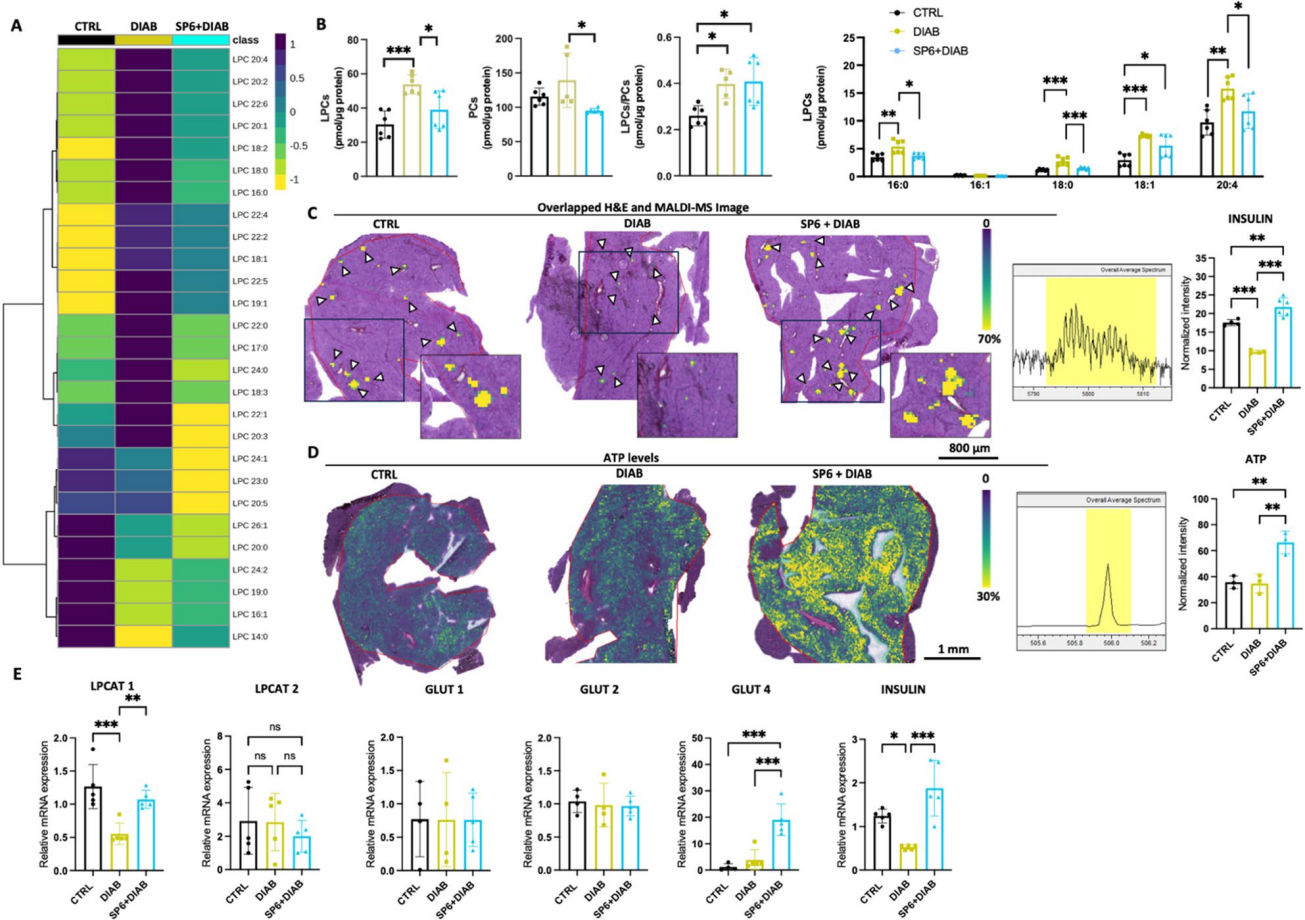
SP6 attenuates the dysregulation of LPC levels and restores insulin secretion by modulating LPCAT1 and GLUT4 in the diabetic pancreas.** A** LC-MS/MS analysis. Heatmaps highlighting the quantitative differential levels of LPC and PC in mice pancreas. **B** Quantification by mass spectrometric analysis of LPC, PC, total LPC/PC, and the most relevant LPCs in mice pancreas. **C** MALDI-MSI images showing spatial distribution of insulin secretion in pancreas following high glucose or SP6 pretreatment, compared to the control group. Overlay images of H&E staining and MALDI-MSI data at m/z 5805 are shown, with white arrows indicating pancreatic islets. Spatial resolution, 50 μm. Data were normalized to the total ion count (TIC). Scale bar = 800 μm. Inset scale bar = 200 μm. The color scale bar corresponds to the percentage of maximum intensity. Bar charts graphs show the differences between the groups in the regions of interest. **D** MALDI-MSI images showing spatial distribution of ATP at m/z 505.99 secretion in pancreas following high glucose or SP6 pretreatment, compared to the control group, Scale bar = 1 mm. Spatial resolution, 50 μm. Data were normalized to the total ion count (TIC). The color scale bar corresponds to the percentage of maximum intensity. Bar charts graphs show the differences between the groups in the regions of interest. **E** Relative mRNA expression is given as relative quantification (RQ = 2-ΔΔCT) values of LPCAT1. 2-ΔΔCT values of the first controls were normalized to 1

**Fig. 6:**
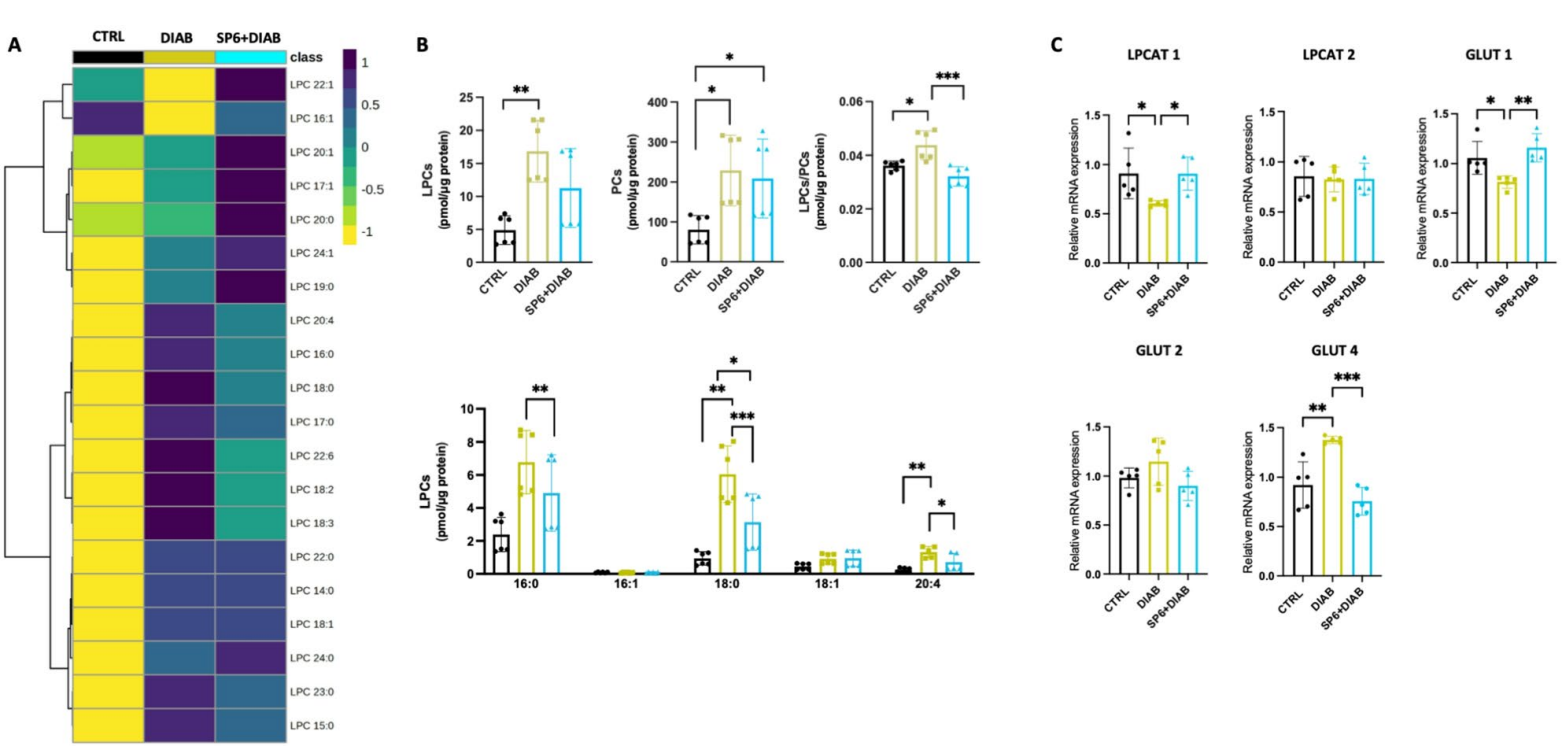
SP6 attenuates the dysregulation of LPC levels and modulates LPCAT1 and GLUT4 in the diabetic liver. **A** LC-MS/MS analysis. Heatmap highlighting the differential levels of LPC and PC in the liver of mice. **B** Quantitative profile of LPC, PC, total LPC/PC, and of the most relevant LPCs in mice liver. **C** Relative mRNA expression is given as relative quantification (RQ = 2^−ΔΔCT^) values of LPCAT1. 2^−ΔΔCT^ values of the first controls were normalized to 1

To elucidate the restoration of insulin secretion in diabetic mice treated with SP6, we investigated the LPC/PC axis in the liver and in the pancreas. Lipidomic profiling of pancreatic tissue demonstrated a significant elevation in total LPC levels in diabetic conditions, characterized by specific increases in LPC 16:0, 18:0, and 20:4 **(**Fig. 5A–B**).** Notably, LPC 14:0, containing myristic acid, exhibited a substantial reduction. A comparable pattern of LPC dysregulation was observed in hepatic tissue **(**Fig. 6A–B**)**. Conversely, administration of SP6 effectively mitigated these alterations, restoring LPC levels to levels comparable to those of the control group. In the pancreas, this normalization was particularly pronounced for LPC species containing one or more double bonds, relative to saturated LPCs **(**Figs. 5A–B and Supplementary Fig. S4).

By using a MALDI-MSI method, we gained valuable insights into the effects of SP6 treatment on insulin secretion. As shown in Fig. 5C, insulin levels were significantly lower in pancreatic islets of diabetic patients than in the control group, indicating a marked reduction in insulin secretion. Surprisingly, SP6 pretreatment stabilized insulin levels to those observed in the control group, suggesting that SP6 protects the pancreatic gland from hyperglycemia-induced damage. Coherently, the detection of ATP levels, necessary signaling for insulin secretion, revealed a massive increase in SP6-treated diabetic mice compared to vehicle-treated diabetic controls (Fig. 5D). Concomitantly, LPCAT1 mRNA expression was reduced in diabetic mice but preserved in SP6-treated animals in association with enhanced expression of GLUT4 transcripts, the primary glucose transporter in pancreatic β-cells (Fig. 5E).

Similarly to the observations in vivo in the diabetic animal model (Fig. 5), we detected a significant decrease in ATP levels in β-TC6 cells exposed to hyperglycemic conditions (Supplementary Fig. S5A). This reduction reflects impaired energy metabolism, consistent with the pancreatic alterations observed by MALDI-imaging (Fig. 5C). Importantly, treatment with SP6 significantly prevented the decline in ATP levels. However, this protective effect was markedly diminished in β-TC6 cells with LPCAT1 silencing, resulting in the loss of SP6’s beneficial action. Concomitantly, high glucose exposure induced a significant decrease in insulin release from β-TC6 cells. SP6 pretreatment preserved insulin secretion under hyperglycemic stress (Supplementary Fig. S5B-C). Notably, insulin release was significantly reduced in LPCAT1-deficient cells treated with SP6 under high glucose, indicating the necessity of LPCAT1 for SP6 efficacy. Mechanistically, hyperglycemia and LPCAT1 silencing led to significant downregulation of LPCAT1 expression and Akt phosphorylation at Ser473 (Supplementary Fig. S5D), a key signaling event for insulin secretion [33].

In contrast, the effects of SP6 in the liver were more diverse across the LPC molecular species, showing no clear dependence on chain length or degree of unsaturation (Fig. 6A–B and Supplementary Fig. S4). Regardless of these factors, we also observe in the liver a preservation of the transcriptional expression of LPCAT1, GLUT1, and GLUT4, in contrast to the dysregulation observed in diabetic conditions. This demonstrates SP6’s ability to preserve the homeostatic regulation of these glucose transporters, maintaining their expression at levels comparable to controls (Fig. 6C).

### Protective role of SP6 on LPCAT1 and GLUTs expression across peripheral and central tissues in a mouse model of type 2 diabetes

At the transcript level in skeletal muscle, SP6 preserves LPCAT1 and LPCAT3 expression at levels comparable to controls, whereas LPCAT2 remains unchanged. SP6 treatment also preserves cytoprotective API5 expression and maintains GLUT1 and GLUT4 expression levels, which were altered in diabetic animals (Supplementary Fig. S6A).

In adipose tissue, SP6 preserves LPCAT1 and LPCAT3, and API5 expression without altering LPCAT2 levels. Regarding the glucose transporter, SP6 preserves physiological levels of GLUT4, whereas GLUT2 expression is unaffected (Supplementary Fig. S6B).

Although the in vitro PAMPA-blood-brain-barrier (BBB) assay suggests that SP6 does not cross the BBB by passive diffusion, which would require an ad-hoc in vivo experiment to employ all physiologically active transport mechanisms, rt-PCR performed at the end of in vivo treatment demonstrates that in brain of SP6-treated mice, LPCAT1, API5, GLUT1 and GLUT4 expression levels are preserved, in contrast to that observed in diabetic mice. Conversely, it prevents the increase in GLUT2 levels observed in diabetic mice, maintaining GLUT2 levels at the level observed in control mice (Supplementary Figs. S6C and S6E). Mechanistically, SP6 led to a significant increase in Akt phosphorylation at Ser473 (Supplementary Fig. S5D).

## Discussion

Diabetes remains a major clinical and scientific challenge [34, 35], where lipid reprogramming, involving complex metabolic abnormalities, plays a critical role in disease progression by affecting cellular signaling, vascular health, and tissue repair [36–41]. Among these lipid changes, the disruption of the LPC/PC balance may affect glucose homeostasis, contributing to endothelial dysfunction and impaired wound healing, hallmark complications of diabetes [26, 42].

The present study aims to elucidate the mechanism of action of the peptide SP6 under diabetic conditions, focusing on its capability to preserve lipid homeostasis and protect endothelial and metabolic functions by preserving LPCAT1 expression. Furthermore, this study identifies lysolecithin reprogramming as a key mechanism driving hyperglycemia-induced endothelial dysfunction and systemic metabolic disturbances characteristic of T2DM. By investigating the remodeling of LPC/PC ratios within distinct subcellular compartments of endothelial and metabolic tissues, we demonstrate that an imbalance in this axis weakens membrane integrity, impairs endothelial function, and affects glucose homeostasis.

By leveraging the SP6 (GIVAGDVTPI) peptide [20], an orally hydrolysis-resistant derivative of *Spirulina platensis* [43–45], we discovered its ability to coordinate lipid reprogramming, maintain endothelial integrity, and support systemic glucose homeostasis.

There is compelling evidence linking several protein frameworks, such as VEGF, cadherin, GLUT transporters, API5, and LPCAT1, through their reliance on membrane lipid composition and dynamics, cell survival, and homeostasis under metabolic stress [27, 29, 46–53]. Our study shows that SP6, by preserving LPCAT1 levels under high-glucose conditions, maintains membrane stability and the balance of LPC/PC, which, in turn, supports API5, VEGF, and GLUT1 expression, ensuring endothelial integrity and metabolic adaptation in diabetes.

To translate these in vitro findings into an in vivo setting, we employed a mouse model of T2DM induced by a combined high-fat diet (HFD) and low-dose streptozotocin (STZ) administration. This model effectively recapitulates the main pathophysiological features of human T2DM, including endothelial dysfunction, insulin resistance, hypoinsulinemia, and dyslipidemia. The HFD promotes obesity and insulin resistance, while low-dose STZ partially impairs pancreatic β-cell function, together reproducing the progressive metabolic decline typical of human T2DM, thus providing a relevant model for studying endothelial and metabolic complications in a physiologically meaningful context [54, 55]. In this model, preventive administration of SP6 effectively mitigates hyperglycemia and counteracts the alterations in plasma LPC profiles, notably inhibiting the pathological increase of LPC 20:4, a proinflammatory arachidonic acid-containing lipid associated with immune regulation.

Given the endothelium’s critical role in diabetes pathophysiology [56] and as the already characterized target of SP6’s action [20], we assessed the role of the peptide within metabolically pivotal diabetic organs, such as the pancreas and liver [57–59], as well as in muscle, adipose tissue, and brain, recognized as key targets of metabolic dysfunction in diabetes [60]. SP6 exerted broad protective effects by enhancing insulin secretion, promoting glucose uptake via transporter regulation, and preserving endothelial barrier function. Mechanistically, these effects were tightly associated with the preservation of LPCAT1 expression at the transcript level and the control of aberrant lysolecithin reprogramming within these tissues.

Using MALDI imaging, we confirmed that SP6 enhanced pancreatic insulin secretion, likely by increasing ATP production and preserving GLUT4 transcripts, key promoters of glucose-stimulated insulin release in β-cells [61, 62]. This multisystemic protective pattern implicates LPCAT1 as a pivotal effector of SP6’s mechanism, whose mRNA expression levels are preserved, suggesting the decapeptide as a novel promising preventive strategy to counteract the aberrant lysolecithin remodeling pathway in diabetic conditions.

## Limitations

Our preliminary data demonstrate that fluorescein-labeled SP6 localizes to both cytosolic and membrane compartments of endothelial cells and activates the Akt signaling pathway (**Supplementary Fig. S7**); however, the precise molecular mechanisms by which SP6 exerts its biochemical effects remain incompletely characterized. This highlights the need for further studies, including molecular modeling, chemical proteomics, and biochemical validations, to identify SP6 interactors and fully elucidate its signaling pathways.

Regarding the in vivo validation, while our data provide valuable insights into SP6’s effects on LPCAT1-mediated lipid remodeling and endothelial function, a potential limitation is the absence of in vivo conditional LPCAT1 knockout (KO) mouse models, especially endothelial-specific ones, which would ideally enable us to examine the role of SP6/LPCAT1 in vivo directly. Therefore, future studies should focus on generating this animal model for the first time.

## Conclusions

This study provides the first evidence that an orally administered peptide from *Spirulina platensis*, generated via simulated gastro-intestinal digestion, exerts antidiabetic effects by acting at multiple systemic levels through lysolecithin reprogramming mediated by the LPCAT1 enzyme. This evidence establishes a basis for future studies investigating the preventive potential of SP6 in individuals at risk for type 2 diabetes mellitus.

Furthermore, our results extend beyond diabetes, suggesting that dysregulation of the LPC/PC machinery may represent a common pathogenic factor across various chronic diseases. The development of preventive natural compound-based interventions, such as SP6, offers promising avenues to preserve metabolic and endothelial health and to mitigate the risk of chronic complications.

## Supplementary Information

Supplementary Material 1.

## Data Availability

The authors declare that the data supporting the findings of this study are available within the paper and its Supplementary Information. Other source data related to the study are available from the corresponding author upon reasonable request.
