# Supplementary material for: Lysolecithin reprogramming via LPCAT1 modulation restores endothelial function and prevents diabetes-associated dysmetabolism
Source: Cardiovasc Diabetol. 2026 Jan 18;25:45. doi: 10.1186/s12933-025-03053-4 (PMC12903598; doi:10.1186/s12933-025-03053-4)
Supplement: Supplementary file 1 — Supplementary Material 1. [file 12933_2025_3053_MOESM1_ESM.pdf]

SUPPLEMENTARY MATERIALS

Lysolecithin Reprogramming via LPCAT1 Modulation Restores Endothelial Function and Prevents Diabetes-Associated Dysmetabolism

Eduardo Maria Sommella[1], Concetta Iside[2], Paola Di Pietro[2], Fabrizio Merciai[1], Emanuela Salviati[1], Marina Sala[1], Angela Carmelita Abate[2], Antonio Damato[3], Massimiliano De Lucia[3], Eleonora Venturini[3], Valeria Prete[2], Francesca Picone[2], Paolo Poggio[4,5], Pasquale Mone[6,7], Michele Ciccarelli[2], Gaetano Santulli[8,9], Pietro Campiglia[1], Carmine Vecchione[2,3], Albino Carrizzo[2,3]

[1] Department of Pharmacy, University of Salerno, Fisciano, SA, 84084, Italy

[2] Department of Medicine, Surgery and Dentistry, "Scuola Medica Salernitana" University of Salerno, Baronissi, Italy;

[3] Vascular Physiopathology Unit, IRCCS Neuromed, Pozzilli, Italy;

[4] Centro Cardiologico Monzino IRCCS, Milan, Italy.

[5] University of Milan, Department of Biomedical, Surgical, and Dental Sciences, Milan, Italy.

[6] University of Molise, Department of Medicine and Health Sciences "Vincenzo Tiberio", Campobasso, Italy.

[7] Casa di Cura Montevergine, Mercogliano, Italy.

[8] Department of Medicine (Cardiology), Albert Einstein College of Medicine, New York City, NY, USA.

[9] International Translational Research and Medical Education (ITME) Consortium, Academic Research Unit, and Department of Advanced Biomedical Sciences, "Federico II" University, Naples, Italy.

Correspondence to: Albino Carrizzo, Università degli Studi di Salerno, Via S. Allende–84081, Baronissi (SA), Italy; IRCCS Neuromed, Vascular Physiopathology Unit, 86077, Pozzilli, Italy; e-mail : acarrizzo@unisa.it

SUPPLEMENTAL MATERIAL

Supplementary Figure S1

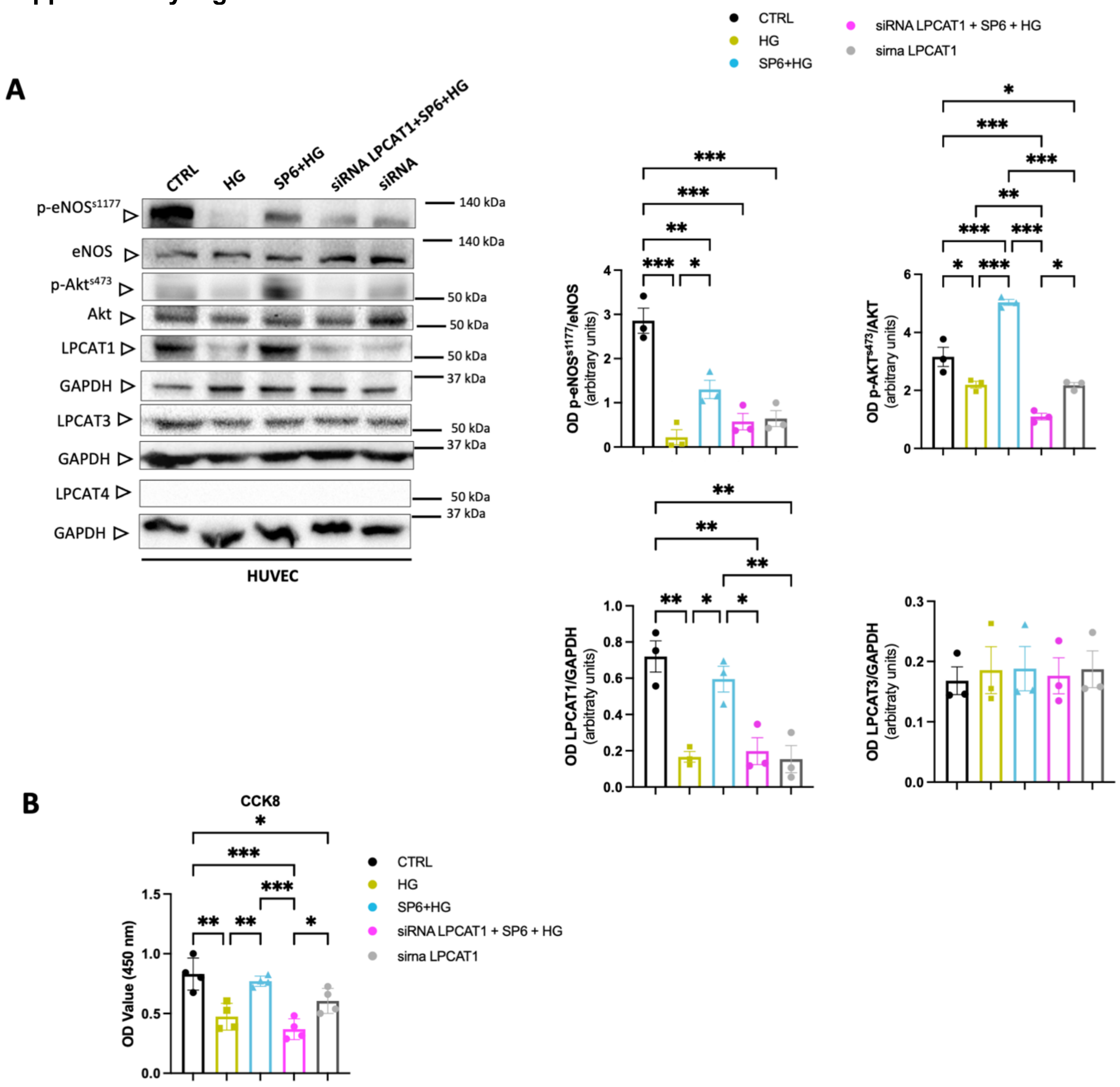

Fig. S1. SP6 protects endothelial cell viability and activates the AKT/eNOS pathway under high glucose via an LPCAT1-dependent mechanism. A) Representative immunoblot analyses evaluating protein levels from HUVECs transfected with LPCAT1 siRNA or control siRNA. Cells were pre-treated with SP6 (100 µg/mL) for 2h and subsequently exposed to high glucose (HG, 30 mM) for 48 h. LPCAT1 silencing efficiency is shown in the corresponding panel. GAPDH was used as a loading control. Densitometric ratios (p-AKT/AKT and p-eNOS/eNOS) are shown alongside blots, expressed as fold change relative to the normoglycemic control. **B**) CCK-8 Cell Viability Assay. This assay was performed following the wound-healing assay on cells subjected to the same sequential exposure (SP6 pre-treatment followed by HG for 48 h). Cell viability was assessed by measuring the absorbance at 450 nm after a 2-hour incubation with the CCK-8 reagent.

Supplementary Figure S2

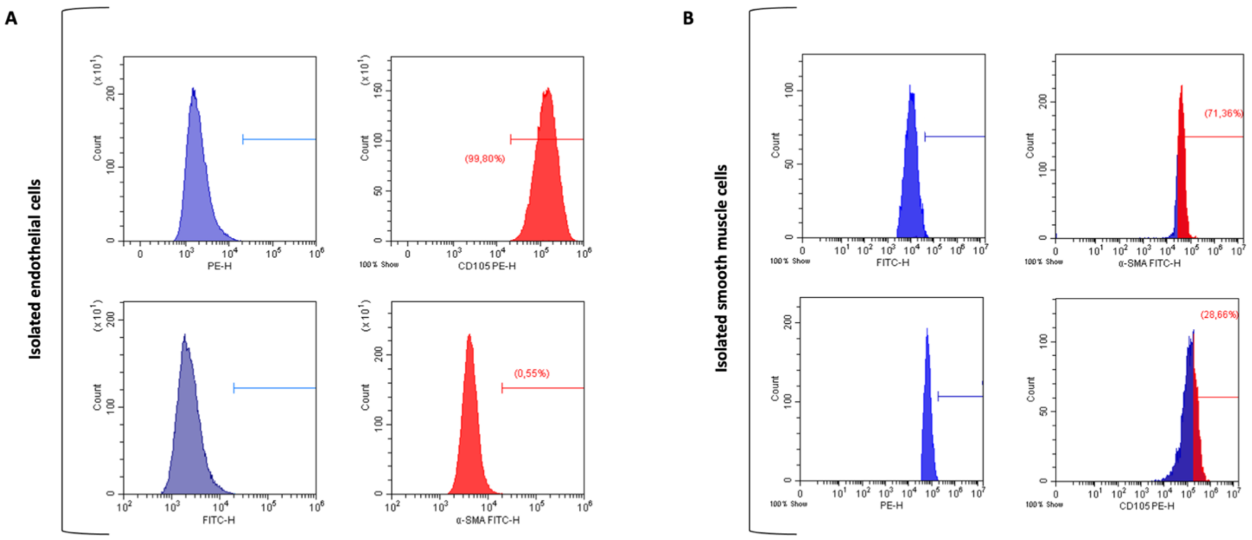

Fig. S2. Cytofluorimetric analysis of mouse isolated mesenteric artery ECs and smooth muscle cells. A) As a negative control for the fluorescence channels used, an aliquot of unstained cells was acquired on DxFLEX flow cytometer to identify any autofluorescence signals. The same cells were then stained with CD105 PE (Beckman Coulter #B92442) and α-SMA FITC (Abcam # ab5694) (cell permeabilization was performed using the PerFix-nc kit, Beckman Coulter). As shown in Figure A, a positive signal for CD105 (99.80%) and a negative signal for α-SMA were detected. **B**) Cytofluorimetric analysis of mouse mesenteric artery smooth muscle cells (SMCs). As a negative control for the fluorescence channels used, an aliquot of unstained cells was acquired on DxFLEX flow cytometer Beckman Coulter #B92442) to identify any autofluorescence signals. The same cells were then stained with CD105 PE (Beckman Coulter) and α-SMA FITC (Abcam #ab5694) (cell permeabilization was performed using the PerFix-nc kit, Beckman Coulter). As shown in Figure B, a positive signal for α-SMA (71.36%) and a CD105-positive signal detected in 28.66% of the cells were observed.

Supplementary Figure S3

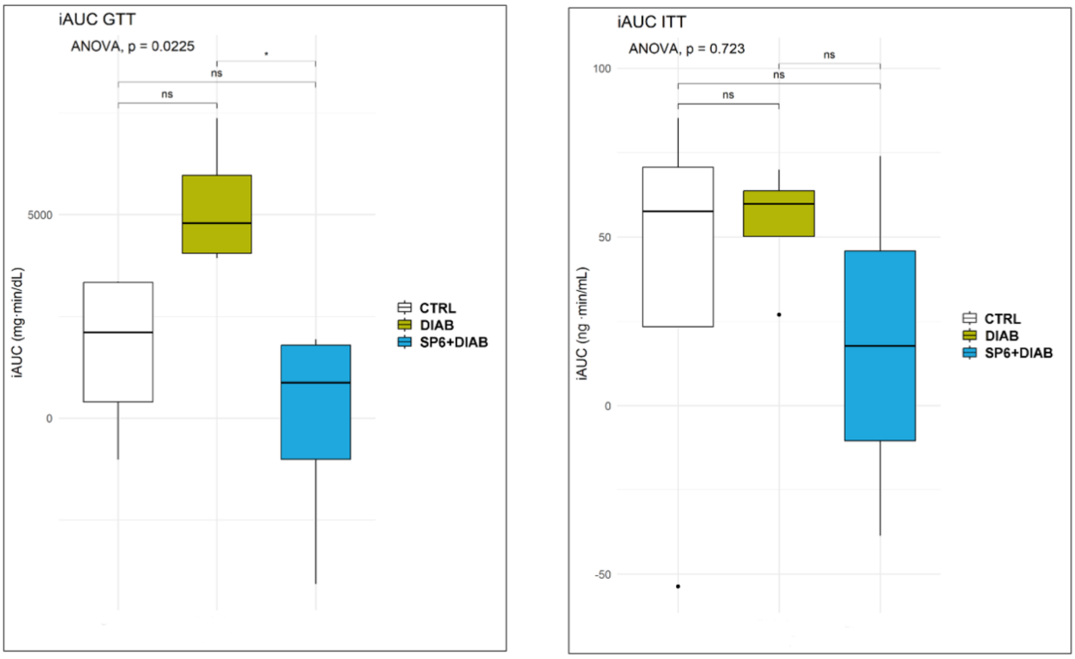

Fig. S3. Distribution of incremental AUC (iAUC) values for glucose tolerance test (GTT) and insulin tolerance test (ITT) across the three experimental groups. Blood glucose values were measured at 0, 30, 60, 90 and 120 minutes during GTT and ITT. For each animal, the iAUC was computed by subtracting the baseline glucose value (time 0) from each subsequent measurement prior to curve integration, thereby quantifying the net glycemic excursion above basal levels. Boxplots represent the distribution of iAUC values across groups for GTT e ITT. ($p > 0.05$).

Supplementary Figure S4

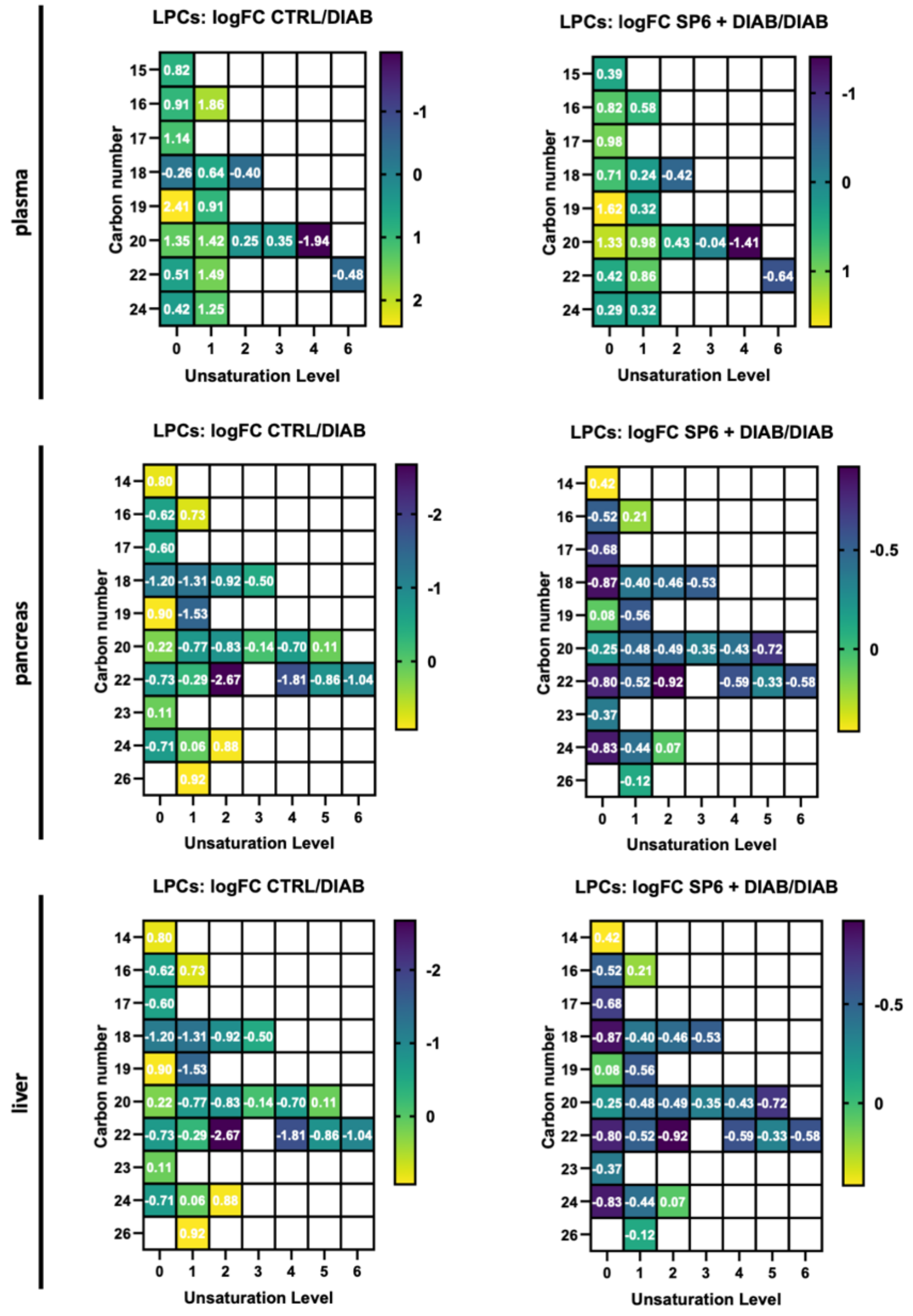

Fig. S4. SP6 preserves LPCs composition in plasma, pancreas, and liver of diabetic mice. Heatmaps showing the Log2FC ratios in plasma, pancreas, and liver tissues collected from control (CTRL), diabetic mice (DIAB), and diabetic mice pretreated with SP6 (SP6 + DIAB) reporting carbon number with the lysolecithin saturation levels.

Supplementary Figure S5

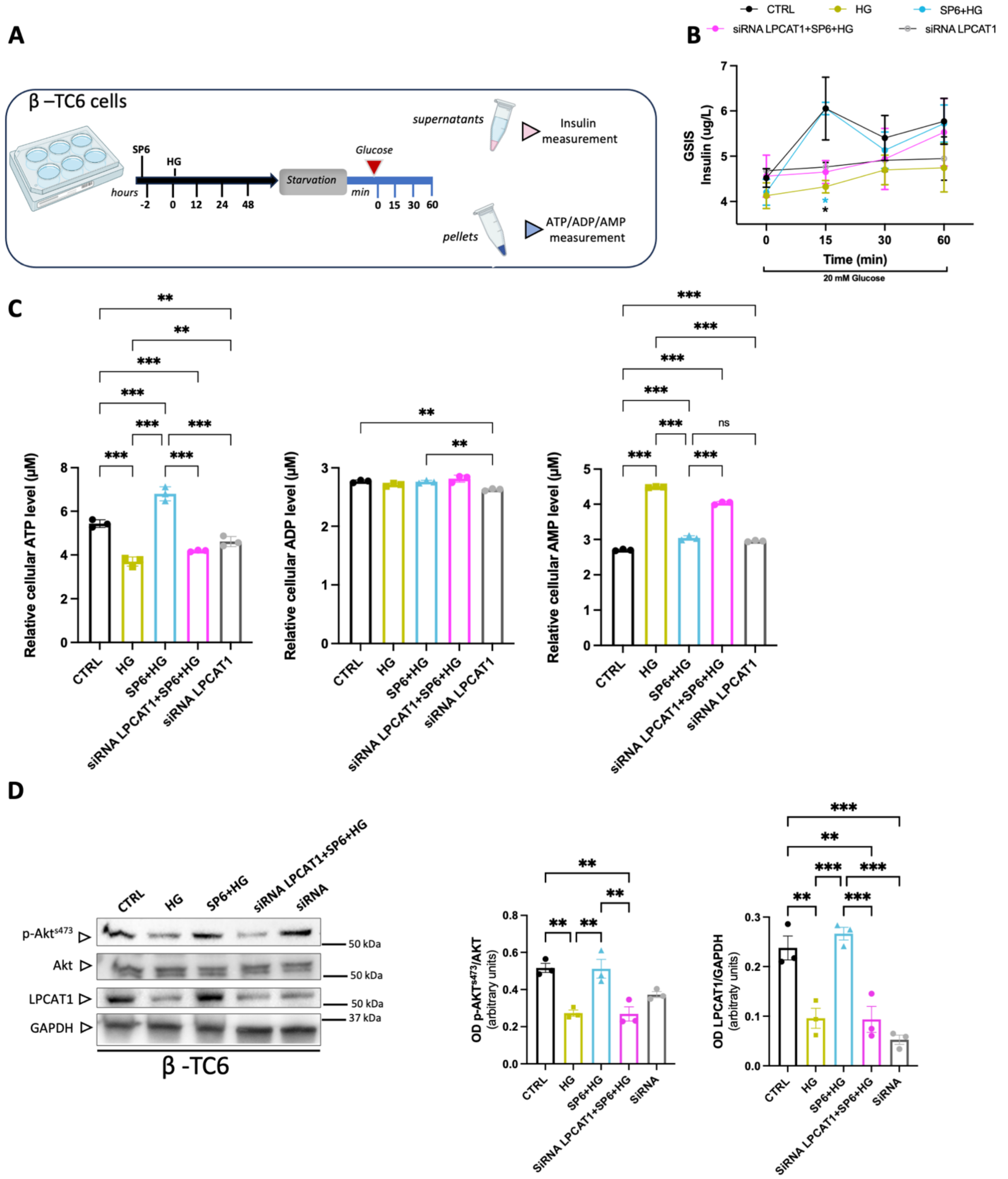

Fig. S5. Glucose-stimulated insulin secretion (GSIS) and intracellular ATP levels in β-TC-6 cells.
A) Glucose-stimulated insulin secretion (GSIS) experimental setting. β-TC-6 cells were transfected with LPCAT1 siRNA or control siRNA, pre-treated with SP6 (100 μg/mL, 2 h), and then exposed for 48 h to either euglycemic conditions (5.5 mM glucose) or high glucose conditions (30 mM glucose). After treatment, cells were starved for 6 h and stimulated with 25 mM glucose for 1 h. **B)** Conditioned media were collected at 0, 15, 30, and 60 minutes, and insulin secretion was quantified by ELISA. Time 0 represents insulin levels after starvation before the addition of glucose (20mM). **C)** Intracellular ATP quantification by LC–MS/MS. Using the same experimental conditions as in panel A, intracellular ATP, ADP, AMP levels were measured by targeted LC–MS/MS and normalized to total protein content. **D)** Representative immunoblot analyses of 3 independent experiments evaluating protein levels from β-TC6 cells transfected with LPCAT1 siRNA or control siRNA. Cells were pre-treated with SP6 (100 μg/mL) for 2 hours and subsequently exposed to high glucose (HG, 30 mM) for 48 hours. The efficiency of LPCAT1 silencing is shown in the corresponding panel. GAPDH was used as a loading control. Densitometric ratios (p-AKT/AKT) are shown alongside the blot and expressed as fold change relative to the normoglycemic control. Data are mean±S.E.M. *P<0.05, **P<0.01 and ***P<0.001.

Supplementary Figure S6

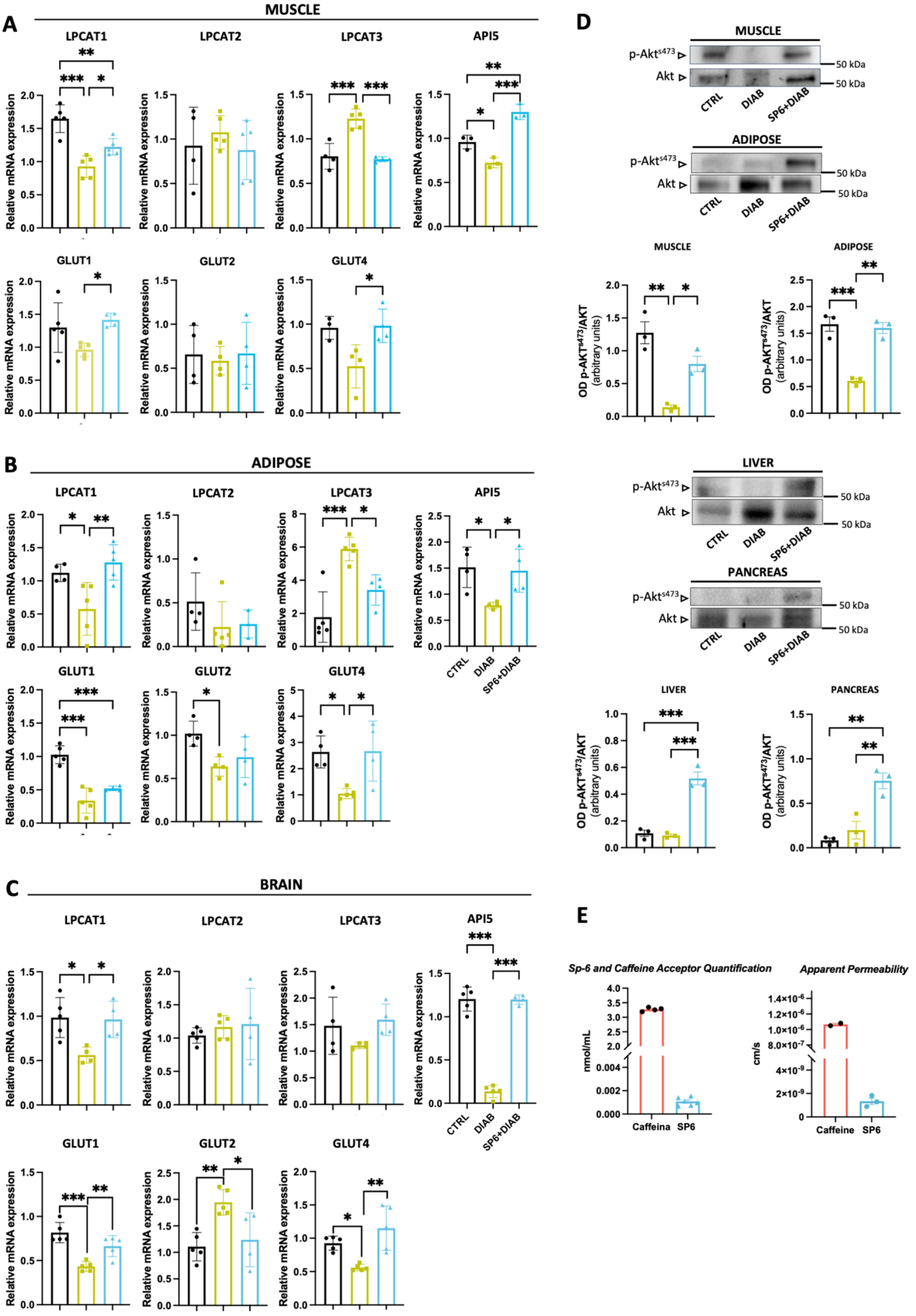

Fig. S6. SP6 exerts coordinated transcriptional effects across multiple metabolic organs.
A) Relative mRNA expression of LPCAT1, LPCAT2, LPCAT3, API5, GLUT1, GLUT2, and GLUT4 was quantified by RT-qPCR in skeletal muscle (**A**), adipose tissue (**B**), and brain (**C**). Gene expression levels are presented as relative quantification (RQ =

2^−ΔΔCT). **D**) Representative immunoblot analyses showing phosphorylated AKT (p-AKTs473) and total AKT in skeletal muscle, adipose tissue, liver, and pancreas from control (CTRL), diabetic (DIAB), and SP6-treated diabetic mice (SP6+DIAB). Corresponding densitometric ratios (p-AKT/AKT) are reported below each blot. **E**) PAMPA assay assessing the passive permeability of SP6 across an artificial lipid membrane. Caffeine was used as a high-permeability reference compound to validate assay performance.

Supplementary Figure S7

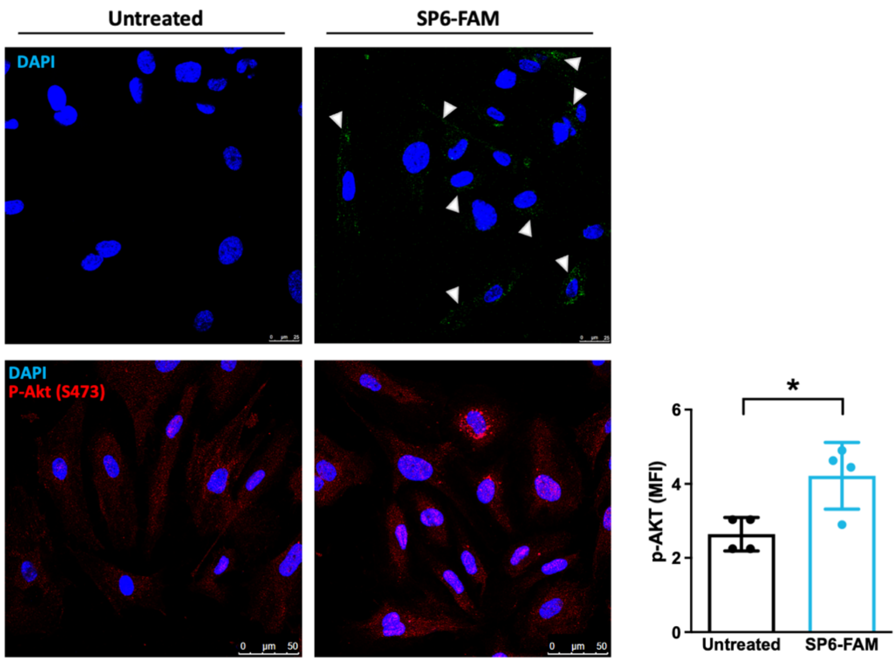

Fig S7. Confocal representative image showing the intracellular localization of SP6 in human endothelial cells. Cells were treated with SP6-FAM (100 mg/mL) for 60 min. DAPI was used to mark nuclei (blue). (***upper***) Confocal representative images of cells treated with SP6-FAM marked in green. (***bottom***) Confocal representative images of phospho-AKT expression (red) in human endothelial cells in basal condition (untreated) or with SP6-FAM (100 mg/mL) for 60 min; DAPI was used to mark nuclei (blue). (***right***) Histograms showing the significant increase of phospho-AKT expression (mean fluorescent intensity, MFI) in SP6-treated endothelial cells compared with the untreated group. * p<0.05

Supplementary table 1

List of identified lysophosphatidylcholines and phosphatidylcholines. Abbreviations: RT, retention time; m/z meas, mass to charge measured; Mass meas., measured mass; CCS, cross collisional section; Mob., mobility; LPC, lysophosphatidylcholine; PC, phosphatidylcholine. Primary ion has been used for quantification

Cytosolic fraction extract

RT [min]	Mob. 1/K0	CCS (Å²)	m/z meas.	M meas.	Ions	Lipid	Molecular Formula
0,42	1,11	228,6	482,32415	481,31687	[M+H]+	LPC 15:0	C23H48NO7P
0,46	1,123	231,1	496,34055	495,33327	[M+H]+	LPC 16:0	C24H50NO7P
0,39	1,112	228,9	494,32428	493,317	[M+H]+	LPC 16:1	C24H48NO7P
0,62	1,166	239,7	524,37142	523,36414	[M+H]+	LPC 18:0	C26H54NO7P
1,63	1,374	280,5	706,53776	705,53059	[M+H]+, [M+Na]+	PC 14:0_16:0	C38H76NO8P
1,56	1,377	281	718,53535	717,52807	[M+H]+	PC 15:0_16:1	C39H76NO8P
1,86	1,407	287,1	734,56932	733,56195	[M+H]+, [M+Na]+, [M+K]+	PC 16:0_16:0	C40H80NO8P
2,1	1,438	293,1	762,60035	761,59307	[M+H]+	PC 16:0_18:0	C42H84NO8P
1,89	1,424	290,3	760,58525	759,57803	[M+H]+, [M+Na]+, [M+K]+	PC 16:0_18:1	C42H82NO8P
1,53	1,381	281,8	730,53808	729,5308	[M+H]+	PC 16:1_16:1	C40H76NO8P
1,71	1,408	287,1	758,56934	757,56233	[M+H]+, [M+Na]+	PC 16:1_18:1	C42H80NO8P
2,13	1,453	296,1	788,61602	787,60902	[M+H]+, [M+Na]+	PC 18:0_18:1	C44H86NO8P
1,92	1,441	293,6	786,6005	785,59346	[M+H]+, [M+Na]+, [M+K]+	PC 18:1_18:1	C44H84NO8P
1,41	1,346	275,1	678,50661	677,49933	[M+H]+	PC 28:0	C36H72NO8P
1,47	1,363	278,3	704,52198	703,5147	[M+H]+	PC 30:1	C38H74NO8P
1,67	1,391	283,8	732,55337	731,54756	[M+H]+, [M+Na]+	PC 32:1	C40H78NO8P
2,01	1,438	293,2	774,60054	773,59326	[M+H]+	PC 35:1	C43H84NO8P
2,34	1,463	298	790,63196	789,62469	[M+H]+	PC 36:0	C44H88NO8P
1,75	1,433	292,1	784,58535	783,57807	[M+H]+	PC 36:3	C44H82NO8P
2,03	1,453	296	800,61632	799,60904	[M+H]+	PC 37:2	C45H86NO8P
2,35	1,476	300,6	816,64729	815,64002	[M+H]+	PC 38:1	C46H90NO8P
2,15	1,47	299,4	814,63258	813,62531	[M+H]+	PC 38:2	C46H88NO8P
1,97	1,459	297,1	812,61587	811,6086	[M+H]+	PC 38:3	C46H86NO8P
2,56	1,507	306,6	844,68006	843,67279	[M+H]+	PC 40:1	C48H94NO8P
2,2	1,485	302,3	840,64943	839,64215	[M+H]+	PC 40:3	C48H90NO8P
2,57	1,523	309,9	870,69569	869,68841	[M+H]+	PC 42:2	C50H96NO8P

Membrane fraction extract

RT [min]	Mob. 1/K0	CCS (Å²)	m/z meas.	M meas.	Ions	LongName	Molecular Formula
0,47	1,123	231,2	496,33963	495,33263	[M+H]+, [M+Na]+	LPC 16:0	C24H50NO7P
0,4	1,11	228,5	494,32387	493,31673	[M+H]+, [M+Na]+	LPC 16:1	C24H48NO7P
0,35	1,097	225,9	492,30876	491,30148	[M+H]+	LPC 16:2	C24H46NO7P
0,44	1,126	231,6	508,33992	507,33265	[M+H]+	LPC 17:1	C25H50NO7P
0,62	1,167	239,8	524,3709	523,36402	[M+H]+, [M+Na]+, [M+K]+	LPC 18:0	C26H54NO7P
0,49	1,139	234,1	522,35509	521,34792	[M+H]+, [M+Na]+	LPC 18:1	C26H52NO7P
0,41	1,121	230,4	520,33937	519,33225	[M+H]+, [M+Na]+	LPC 18:2	C26H50NO7P
0,86	1,202	246,8	552,40208	551,3948	[M+H]+	LPC 20:0	C28H58NO7P
0,64	1,18	242,1	550,38637	549,3791	[M+H]+	LPC 20:1	C28H56NO7P
0,47	1,155	237,2	546,35474	545,34746	[M+H]+	LPC 20:3	C28H52NO7P
0,4	1,131	232,3	544,33944	543,33033	[M+H]+, [M+Na]+	LPC 20:4	C28H50NO7P
0,88	1,213	248,7	578,41788	577,4106	[M+H]+	LPC 22:1	C30H60NO7P
0,7	1,19	244	576,40237	575,39509	[M+H]+	LPC 22:2	C30H58NO7P
0,57	1,179	241,8	574,38643	573,37915	[M+H]+	LPC 22:3	C30H56NO7P
0,41	1,149	235,7	570,35499	569,34772	[M+H]+	LPC 22:5	C30H52NO7P
1,09	1,298	265,9	608,46638	607,45911	[M+H]+	LPC 24:0	C32H66NO7P
1,13	1,248	255,5	606,44904	605,44176	[M+H]+	LPC 24:1	C32H64NO7P
0,93	1,227	251,2	604,43397	603,42669	[M+H]+	LPC 24:2	C32H62NO7P
1,31	1,327	271,5	636,49627	635,48899	[M+H]+	LPC 26:0	C34H70NO7P
1,36	1,281	262,2	634,48061	633,47333	[M+H]+	LPC 26:1	C34H68NO7P
0,99	1,244	254,6	630,44949	629,44221	[M+H]+	LPC 26:3	C34H64NO7P
1,4	1,346	275	678,50782	677,49999	[M+H]+, [M+Na]+	PC 14:0_14:0	C36H72NO8P
1,24	1,332	272,2	676,49062	675,48334	[M+H]+	PC 14:0_14:1	C36H70NO8P
1,52	1,357	277,2	692,5222	691,51492	[M+H]+	PC 14:0_15:0	C37H74NO8P
1,63	1,376	280,9	706,53871	705,53107	[M+H]+, [M+Na]+	PC 14:0_16:0	C38H76NO8P
1,46	1,365	278,7	704,52337	703,5161	[M+H]+	PC 14:0_16:1	C38H74NO8P
1,35	1,355	276,8	702,50722	701,5001	[M+H]+, [M+Na]+	PC 14:1_16:1	C38H72NO8P
1,78	1,407	287	746,56921	745,56194	[M+H]+	PC 15:0_18:1	C41H80NO8P
1,85	1,407	287	734,57038	733,56264	[M+H]+, [M+Na]+	PC 16:0_16:0	C40H80NO8P
1,67	1,394	284,4	732,55459	731,54731	[M+H]+	PC 16:0_16:1	C40H78NO8P
2,09	1,436	292,8	762,60086	761,593	[M+H]+, [M+Na]+, [M+K]+	PC 16:0_18:0	C42H84NO8P

1,88	1,424	290,4	760,58624	759,57864	[M+H]+, [M+Na]+, [M+K]+	PC 16:0_18:1	C42H82NO8P
2,34	1,476	300,7	816,64746	815,64009	[M+H]+, [M+Na]+	PC 16:0_22:1	C46H90NO8P
1,61	1,435	292,2	806,56885	805,56157	[M+H]+	PC 16:0_22:6	C46H80NO8P
2,88	1,554	316,1	874,72585	873,71857	[M+H]+	PC 16:0_26:0	C50H100NO8P
1,51	1,384	282,3	730,53919	729,53191	[M+H]+	PC 16:1_16:1	C40H76NO8P
1,4	1,376	280,8	728,52195	727,51503	[M+H]+, [M+Na]+, [M+K]+	PC 16:1_16:2	C40H74NO8P
1,71	1,413	288,2	758,57023	757,5628	[M+H]+, [M+Na]+, [M+K]+	PC 16:1_18:1	C42H80NO8P
1,55	1,399	285,3	756,55311	755,54623	[M+H]+, [M+Na]+	PC 16:1_18:2	C42H78NO8P
1,81	1,426	290,6	772,58453	771,57726	[M+H]+	PC 17:1_18:1	C43H82NO8P
1,92	1,468	298,9	824,61633	823,60906	[M+H]+	PC 17:1_22:3	C47H86NO8P
2,13	1,451	295,7	788,61746	787,61018	[M+H]+	PC 18:0_18:1	C44H86NO8P
2,55	1,506	306,6	844,67845	843,67117	[M+H]+	PC 18:0_22:1	C48H94NO8P
1,92	1,443	294	786,60181	785,59399	[M+H]+, [M+K]+, [M+Na]+	PC 18:1_18:1	C44H84NO8P
2,02	1,455	296,5	800,61606	799,60878	[M+H]+	PC 18:1_19:1	C45H86NO8P
1,96	1,46	297,3	812,61599	811,60872	[M+H]+	PC 18:1_20:2	C46H86NO8P
1,83	1,453	295,9	810,59994	809,59266	[M+H]+	PC 18:1_20:3	C46H84NO8P
1,7	1,441	293,6	808,58432	807,57732	[M+H]+, [M+H-H2O]+	PC 18:1_20:4	C46H82NO8P
1,85	1,469	299	836,61537	835,60809	[M+H]+	PC 18:1_22:4	C48H86NO8P
1,71	1,456	296,5	834,6002	833,59292	[M+H]+	PC 18:1_22:5	C48H84NO8P
2,56	1,528	311	870,69403	869,68681	[M+H]+, [M+Na]+	PC 18:1_24:1	C50H96NO8P
2,23	1,513	307,8	866,66275	865,65547	[M+H]+	PC 18:1_24:3	C50H92NO8P
2,88	1,585	322,2	926,75671	925,74943	[M+H]+	PC 18:1_28:1	C54H104NO8P
2,99	1,61	327,1	954,78833	953,78106	[M+H]+	PC 18:1_30:1	C56H108NO8P
2,99	1,593	323,9	928,77278	927,7655	[M+H]+	PC 20:0_26:1	C54H106NO8P
1,43	1,454	296	854,56895	853,56168	[M+H]+	PC 20:4_22:6	C50H80NO8P
2,91	1,621	329,3	978,78783	977,78056	[M+H]+	PC 22:0_28:4	C58H108NO8P
0,98	1,277	261,4	622,44409	621,43681	[M+H]+	PC 24:0_26:5	C32H64NO8P
1,19	1,312	268,4	650,47571	649,46844	[M+H]+	PC 26:0	C34H68NO8P
2,76	1,576	320,3	924,74092	923,73364	[M+H]+	PC 26:1_20:2	C54H102NO8P
1,13	1,322	270,1	674,47543	673,46816	[M+H]+	PC 28:2	C36H68NO8P
1,37	1,349	275,5	690,50699	689,49971	[M+H]+	PC 29:1	C37H72NO8P
1,74	1,389	283,4	720,55351	719,54623	[M+H]+	PC 31:0	C39H78NO8P
1,57	1,377	281,1	718,53763	717,53035	[M+H]+	PC 31:1	C39H76NO8P
1,42	1,371	279,9	716,52245	715,51517	[M+H]+	PC 31:2	C39H74NO8P
1,25	1,359	277,4	726,50654	725,49926	[M+H]+	PC 32:4	C40H72NO8P
1,6	1,394	284,4	744,55345	743,54617	[M+H]+	PC 33:2	C41H78NO8P
1,3	1,381	281,6	752,5219	751,51462	[M+H]+	PC 34:5	C42H74NO8P

2,17	1,449	295,4	776,61641	775,60913	[M+H]+	PC 35:0	C43H86NO8P
2	1,44	293,4	774,6002	773,59293	[M+H]+	PC 35:1	C43H84NO8P
1,55	1,408	287,1	768,55369	767,54642	[M+H]+	PC 35:4	C43H78NO8P
2,33	1,462	297,9	790,63134	789,62406	[M+H]+	PC 36:0	C44H88NO8P
1,02	1,296	265,1	648,45945	647,45218	[M+H]+	PC 36:1	C34H66NO8P
1,74	1,43	291,4	784,58475	783,57782	[M+H]+, [M+Na]+	PC 36:3	C44H82NO8P
1,67	1,426	290,7	782,56843	781,56131	[M+H]+, [M+Na]+	PC 36:4	C44H80NO8P
1,5	1,412	287,8	780,55306	779,54579	[M+H]+	PC 36:5	C44H78NO8P
1,39	1,404	286,1	778,53758	777,53031	[M+H]+	PC 36:6	C44H76NO8P
2,23	1,469	299,3	802,63194	801,62466	[M+H]+	PC 37:1	C45H88NO8P
1,85	1,444	294,3	798,60013	797,59285	[M+H]+	PC 37:3	C45H84NO8P
2,55	1,491	303,6	818,66342	817,65614	[M+H]+	PC 38:0	C46H92NO8P
2,14	1,469	299,2	814,63156	813,62475	[M+H]+, [M+Na]+, [M+K]+	PC 38:2	C46H88NO8P
1,53	1,429	291	806,5688	805,56187	[M+H]+, [M+Na]+	PC 38:6	C46H80NO8P
1,44	1,423	289,8	804,55327	803,54599	[M+H]+	PC 38:7	C46H78NO8P
2,25	1,484	302,2	828,64763	827,64035	[M+H]+	PC 39:2	C47H90NO8P
2,07	1,474	300,1	826,63203	825,62475	[M+H]+	PC 39:3	C47H88NO8P
2,74	1,523	310	846,69472	845,68744	[M+H]+	PC 40:0	C48H96NO8P
2,36	1,497	304,8	842,66263	841,6547	[M+H]+, [M+Na]+	PC 40:2	C48H92NO8P
2,03	1,479	301,1	838,63145	837,6245	[M+H]+, [M+Na]+	PC 40:4	C48H88NO8P
1,91	1,472	299,7	836,61594	835,60866	[M+H]+	PC 40:5	C48H86NO8P
1,84	1,467	298,6	834,59998	833,59271	[M+H]+	PC 40:6	C48H84NO8P
1,63	1,453	295,8	832,58434	831,57706	[M+H]+	PC 40:7	C48H82NO8P
2,74	1,538	313	872,70984	871,70257	[M+H]+	PC 42:1	C50H98NO8P
2,39	1,521	309,4	868,67829	867,67101	[M+H]+	PC 42:3	C50H94NO8P
2,09	1,501	305,3	864,64731	863,64004	[M+H]+	PC 42:5	C50H90NO8P
1,95	1,49	303,2	862,63206	861,62478	[M+H]+	PC 42:6	C50H88NO8P
1,81	1,48	301,1	860,61579	859,60851	[M+H]+	PC 42:7	C50H86NO8P
1,66	1,469	298,9	858,59969	857,59241	[M+H]+	PC 42:8	C50H84NO8P
1,5	1,456	296,4	856,58418	855,5769	[M+H]+	PC 42:9	C50H82NO8P
2,89	1,569	319	900,74122	899,73394	[M+H]+	PC 44:1	C52H102NO8P
2,74	1,557	316,6	898,72562	897,71834	[M+H]+	PC 44:2	C52H100NO8P
2,58	1,547	314,5	896,70944	895,70217	[M+H]+	PC 44:3	C52H98NO8P
2,42	1,54	313,1	894,69386	893,68658	[M+H]+, [M+Na]+	PC 44:4	C52H96NO8P
2,28	1,53	311,2	892,67863	891,67135	[M+H]+	PC 44:5	C52H94NO8P
1,98	1,509	306,8	888,64808	887,6408	[M+H]+	PC 44:7	C52H90NO8P
1,82	1,497	304,4	886,63228	885,625	[M+H]+	PC 44:8	C52H88NO8P
2,82	1,566	318,4	912,74247	911,7352	[M+H]+	PC 45:2	C53H102NO8P
2,46	1,558	316,8	920,70956	919,70228	[M+H]+	PC 46:5	C54H98NO8P
2,2	1,541	313,2	916,67885	915,67157	[M+H]+	PC 46:7	C54H94NO8P

2,89	1,602	325,5	952,77191	951,76463	[M+H]+	PC 48:3	C56H106NO8P
2,8	1,591	323,2	950,75725	949,74997	[M+H]+	PC 48:4	C56H104NO8P
2,66	1,585	322	948,74166	947,73438	[M+H]+	PC 48:5	C56H102NO8P
2,52	1,579	320,8	946,72577	945,7185	[M+H]+	PC 48:6	C56H100NO8P
2,38	1,568	318,6	944,70928	943,70201	[M+H]+	PC 48:7	C56H98NO8P
2,23	1,56	316,9	942,69426	941,68699	[M+H]+	PC 48:8	C56H96NO8P
2,79	1,614	327,9	976,77253	975,76525	[M+H]+	PC 50:5	C58H106NO8P
2,68	1,607	326,4	974,75739	973,75011	[M+H]+	PC 50:6	C58H104NO8P
2,92	1,639	332,8	1004,8042	1003,7969	[M+H]+	PC 52:5	C60H110NO8P
2,81	1,633	331,5	1002,7894	1001,7821	[M+H]+	PC 52:6	C60H108NO8P

Extracellular fraction extract

RT [min]	Mob. 1/K0	CCS (Å²)	m/z meas.	M meas.	Ions	Lipid	Molecular Formula
0,37	1,092	225	468,30856	467,30129	[M+H]+	LPC 14:0	C22H46NO7P
0,42	1,11	228,7	482,3244	481,31712	[M+H]+	LPC 15:0	C23H48NO7P
0,47	1,124	231,2	496,33979	495,33263	[M+H]+, [M+Na]+	LPC 16:0	C24H50NO7P
0,39	1,111	228,6	494,32404	493,31706	[M+H]+, [M+Na]+	LPC 16:1	C24H48NO7P
0,54	1,145	235,5	510,35498	509,34771	[M+H]+	LPC 17:0	C25H52NO7P
0,62	1,167	239,9	524,37091	523,36378	[M+H]+, [M+Na]+	LPC 18:0	C26H54NO7P
0,49	1,139	234,1	522,35529	521,3482	[M+H]+, [M+Na]+	LPC 18:1	C26H52NO7P
0,41	1,118	229,8	520,34002	519,33274	[M+H]+	LPC 18:2	C26H50NO7P
0,85	1,202	246,7	552,40262	551,39534	[M+H]+	LPC 20:0	C28H58NO7P
0,64	1,178	241,8	550,38696	549,37969	[M+H]+	LPC 20:1	C28H56NO7P
0,44	1,134	232,8	546,35525	545,34797	[M+H]+	LPC 20:3	C28H52NO7P
0,4	1,13	232,1	544,33949	543,33124	[M+H]+, [M+Na]+	LPC 20:4	C28H50NO7P
0,41	1,15	235,8	570,35512	569,34798	[M+H]+, [M+Na]+	LPC 22:5	C30H52NO7P
1,13	1,248	255,6	606,44937	605,4421	[M+H]+	LPC 24:1	C32H64NO7P
1,32	1,328	271,8	636,49604	635,48876	[M+H]+	LPC 26:0	C34H70NO7P
1,36	1,283	262,6	634,48055	633,47327	[M+H]+	LPC 26:1	C34H68NO7P
1,47	1,365	278,7	704,52193	703,51514	[M+H]+, [M+Na]+	PC 14:0_16:1	C38H74NO8P
1,87	1,407	287	734,56933	733,56208	[M+H]+, [M+Na]+	PC 16:0_16:0	C40H80NO8P
2,11	1,436	292,8	762,60034	761,59331	[M+H]+, [M+Na]+	PC 16:0_18:0	C42H84NO8P
1,89	1,421	289,8	760,58582	759,57755	[M+H]+, [M+Na]+, [M+K]+	PC 16:0_18:1	C42H82NO8P
1,68	1,421	289,5	782,56866	781,56176	[M+H]+, [M+Na]+	PC 16:0_20:4	C44H80NO8P

1,69	1,439	293,1	808,58431	807,57769	[M+H]+, [M+Na]+, [M+K]+	PC 16:0_22:5	C46H82NO8P
1,62	1,439	293,1	806,56854	805,56149	[M+H]+, [M+Na]+	PC 16:0_22:6	C46H80NO8P
0,47	1,158	238	524,33531	523,32803	[M+H]+	PC 17:0	C25H50NO8P
2,01	1,437	292,9	774,60026	773,59298	[M+H]+	PC 17:0_18:1	C43H84NO8P
1,89	1,444	294,2	798,60051	797,59323	[M+H]+	PC 17:0_20:3	C45H84NO8P
1,73	1,451	295,6	820,58442	819,57714	[M+H]+	PC 17:0_22:6	C47H82NO8P
1,81	1,425	290,4	772,58521	771,57793	[M+H]+	PC 17:1_18:1	C43H82NO8P
2,13	1,451	295,6	788,61734	787,60928	[M+H]+, [M+Na]+, [M+K]+	PC 18:0_18:1	C44H86NO8P
2,01	1,46	297,4	812,61694	811,6092	[M+H]+, [M+Na]+, [M+H-H2O]+, [M+K]+	PC 18:0_20:3	C46H86NO8P
1,91	1,452	295,8	810,60026	809,59314	[M+H]+, [M+K]+, [M+Na]+	PC 18:0_20:4	C46H84NO8P
1,84	1,466	298,4	834,60026	833,5932	[M+H]+, [M+Na]+, [M+K]+	PC 18:0_22:6	C48H84NO8P
1,92	1,438	293	786,60019	785,59321	[M+H]+, [M+K]+, [M+Na]+	PC 18:1_18:1	C44H84NO8P
1,85	1,484	301,9	860,61554	859,60826	[M+H]+	PC 20:1_22:6	C50H86NO8P
1,5	1,459	296,9	856,5834	855,57612	[M+H]+	PC 20:4_22:5	C50H82NO8P
1,41	1,346	275	678,50637	677,49947	[M+H]+, [M+Na]+	PC 28:0	C36H72NO8P
1,24	1,331	272	676,49145	675,48417	[M+H]+	PC 28:1	C36H70NO8P
1,52	1,361	278,1	692,52242	691,51514	[M+H]+	PC 29:0	C37H74NO8P
1,63	1,374	280,6	706,53764	705,53076	[M+H]+, [M+Na]+	PC 30:0	C38H76NO8P
1,68	1,391	283,9	732,55307	731,54634	[M+H]+, [M+Na]+	PC 32:1	C40H78NO8P
1,54	1,384	282,3	730,5377	729,53042	[M+H]+	PC 32:2	C40H76NO8P
1,98	1,424	290,4	748,58491	747,57763	[M+H]+	PC 33:0	C41H82NO8P
1,78	1,404	286,3	746,56925	745,56198	[M+H]+	PC 33:1	C41H80NO8P
1,71	1,406	286,8	758,56881	757,56168	[M+H]+, [M+Na]+	PC 34:2	C42H80NO8P
1,45	1,39	283,4	754,53754	753,53026	[M+H]+	PC 34:4	C42H76NO8P
2,22	1,452	295,9	776,61649	775,60921	[M+H]+	PC 35:0	C43H86NO8P
2,33	1,461	297,8	790,63077	789,62349	[M+H]+	PC 36:0	C44H88NO8P
1,77	1,428	291	784,58431	783,57677	[M+H]+, [M+Na]+, [M+H-H2O]+	PC 36:3	C44H82NO8P
1,53	1,411	287,5	780,55291	779,54563	[M+H]+	PC 36:5	C44H78NO8P
2,25	1,464	298,1	802,63193	801,62466	[M+H]+	PC 37:1	C45H88NO8P
2,05	1,451	295,7	800,61668	799,6094	[M+H]+	PC 37:2	C45H86NO8P
1,79	1,436	292,6	796,58479	795,57751	[M+H]+	PC 37:4	C45H82NO8P

2,56	1,492	303,8	818,6631	817,65582	[M+H]+	PC 38:0	C46H92NO8P
2,36	1,475	300,4	816,64707	815,63979	[M+H]+	PC 38:1	C46H90NO8P
2,15	1,468	299	814,63157	813,62429	[M+H]+	PC 38:2	C46H88NO8P
1,84	1,448	294,9	810,59992	809,59262	[M+H]+, [M+Na]+	PC 38:4	C46H84NO8P
1,44	1,419	289,1	804,55345	803,54617	[M+H]+	PC 38:7	C46H78NO8P
2,13	1,477	300,6	826,63196	825,62469	[M+H]+	PC 39:3	C47H88NO8P
2,03	1,47	299,3	824,61625	823,60897	[M+H]+	PC 39:4	C47H86NO8P
1,8	1,451	295,6	822,60012	821,59285	[M+H]+	PC 39:5	C47H84NO8P
2,57	1,506	306,5	844,67926	843,67199	[M+H]+	PC 40:1	C48H94NO8P
2,37	1,496	304,6	842,66302	841,65574	[M+H]+	PC 40:2	C48H92NO8P
2,25	1,487	302,8	840,64769	839,64042	[M+H]+	PC 40:3	C48H90NO8P
2,07	1,477	300,7	838,63132	837,62405	[M+H]+	PC 40:4	C48H88NO8P
1,92	1,47	299,3	836,61545	835,60783	[M+H]+, [M+K]+, [M+Na]+	PC 40:5	C48H86NO8P
1,64	1,451	295,5	832,58441	831,57713	[M+H]+	PC 40:7	C48H82NO8P
2,03	1,485	302,1	850,63186	849,62459	[M+H]+	PC 41:5	C49H88NO8P
1,96	1,482	301,6	848,61603	847,60875	[M+H]+	PC 41:6	C49H86NO8P
1,74	1,467	298,5	846,59934	845,59207	[M+H]+	PC 41:7	C49H84NO8P
2,76	1,537	312,7	872,70991	871,70264	[M+H]+	PC 42:1	C50H98NO8P
2,57	1,526	310,5	870,69417	869,6869	[M+H]+	PC 42:2	C50H96NO8P
2,43	1,518	308,9	868,67845	867,67117	[M+H]+	PC 42:3	C50H94NO8P
2,24	1,507	306,7	866,66291	865,65563	[M+H]+	PC 42:4	C50H92NO8P
2,15	1,499	304,9	864,64733	863,64005	[M+H]+	PC 42:5	C50H90NO8P
2,08	1,493	303,9	862,63063	861,62335	[M+H]+	PC 42:6	C50H88NO8P
2,75	1,553	315,9	898,72592	897,71864	[M+H]+	PC 44:2	C52H100NO8P

Supplemental Table 2.
List of identified lysophosphatidylcholines and phosphatidylcholines in plasma extract. Abbreviations: RT, retention time; m/z meas, mass to charge measured; Mass meas., measured mass; CCS, cross collisional section; Mob., mobility; LPC, lysophosphatidylcholine; PC, phosphatidylcholine. Primary ion has been used for quantification

**RT [min]**	**Mob. 1/K0**	**CCS (Å²)**	**m/z meas.**	**M meas.**	**Ions**	**Lipid**	**Molecular Formula**
0,43	1,105	227,7	482,3245	481,3172	[M+H]+	LPC 15:0	C23H48NO7P
0,49	1,129	232,3	496,3396	495,3323	[M+H]+	LPC 16:0	C24H50NO7P
0,41	1,103	227	494,324	493,3167	[M+H]+	LPC 16:1	C24H48NO7P
0,54	1,149	236,2	510,3552	509,3491	[M+H]+, [M+Na]+	LPC 17:0	C25H52NO7P
0,64	1,165	239,5	524,3708	523,3635	[M+H]+	LPC 18:0	C26H54NO7P
0,51	1,142	234,8	522,3553	521,3482	[M+H]+, [M+Na]+	LPC 18:1	C26H52NO7P
0,43	1,115	229,2	520,3397	519,3326	[M+H]+, [M+K]+, [M+Na]+	LPC 18:2	C26H50NO7P

0,75	1,182	242,7	538,387	537,3798	[M+H]+	LPC 19:0	C27H56NO7P
0,58	1,159	238,2	536,3714	535,3641	[M+H]+	LPC 19:1	C27H54NO7P
0,88	1,203	246,9	552,4019	551,3949	[M+H]+, [M+Na]+	LPC 20:0	C28H58NO7P
0,66	1,178	241,8	550,3866	549,3796	[M+H]+, [M+Na]+	LPC 20:1	C28H56NO7P
0,54	1,153	236,7	548,371	547,3637	[M+H]+	LPC 20:2	C28H54NO7P
0,46	1,137	233,4	546,3549	545,3476	[M+H]+	LPC 20:3	C28H52NO7P
0,42	1,131	232,3	544,3394	543,3323	[M+H]+, [M+Na]+	LPC 20:4	C28H50NO7P
1,14	1,239	254	580,4335	579,4262	[M+H]+	LPC 22:0	C30H62NO7P
0,9	1,213	248,7	578,4183	577,411	[M+H]+	LPC 22:1	C30H60NO7P
0,4	1,145	234,8	568,3395	567,3323	[M+H]+, [M+Na]+	LPC 22:6	C30H50NO7P
1,37	1,274	260,9	608,4647	607,4578	[M+H]+, [M+Na]+	LPC 24:0	C32H66NO7P
1,15	1,248	255,6	606,4494	605,4422	[M+H]+	LPC 24:1	C32H64NO7P
1,88	1,405	286,7	734,5699	733,5626	[M+H]+	PC 16:0_16:0	C40H80NO8P
1,69	1,388	283,3	732,5534	731,5464	[M+H]+, [M+Na]+	PC 16:0_16:1	C40H78NO8P
2,11	1,438	293,3	762,6001	761,5933	[M+H]+, [M+Na]+	PC 16:0_18:0	C42H84NO8P
1,91	1,42	289,5	760,5858	759,5785	[M+H]+	PC 16:0_18:1	C42H82NO8P
1,73	1,403	286,2	758,5703	757,563	[M+H]+	PC 16:0_18:2	C42H80NO8P
1,69	1,419	289,1	782,5701	781,5628	[M+H]+	PC 16:0_20:4	C44H80NO8P
1,55	1,407	286,7	780,5535	779,5468	[M+H]+, [M+Na]+	PC 16:0_20:5	C44H78NO8P
1,78	1,441	293,6	808,585	807,5777	[M+H]+	PC 16:0_22:5	C46H82NO8P
1,64	1,432	291,7	806,5704	805,5632	[M+H]+	PC 16:0_22:6	C46H80NO8P
1,85	1,421	289,7	772,5848	771,5776	[M+H]+	PC 17:0_18:2	C43H82NO8P
2,14	1,451	295,6	788,6172	787,6093	[M+H]+, [M+Na]+, [M+K]+	PC 18:0_18:1	C44H86NO8P
1,96	1,434	292,2	786,6016	785,5943	[M+H]+	PC 18:0_18:2	C44H84NO8P
2,17	1,464	298,2	814,6314	813,6246	[M+H]+, [M+Na]+	PC 18:0_20:2	C46H88NO8P
2,03	1,455	296,3	812,616	811,6091	[M+H]+, [M+Na]+, [M+H-H2O]+	PC 18:0_20:3	C46H86NO8P
1,93	1,451	295,4	810,6008	809,5936	[M+H]+	PC 18:0_20:4	C46H84NO8P
2,02	1,471	299,5	836,6157	835,6084	[M+H]+	PC 18:0_22:5	C48H86NO8P
1,86	1,465	298,2	834,6012	833,5939	[M+H]+	PC 18:0_22:6	C48H84NO8P
1,77	1,424	290,3	784,5845	783,5773	[M+H]+	PC 18:1_18:2	C44H82NO8P
1,71	1,437	292,7	808,5846	807,5778	[M+H]+, [M+Na]+	PC 18:1_20:4	C46H82NO8P
1,55	1,425	290,3	806,5699	805,5622	[M+H]+, [M+Na]+	PC 18:2_20:4	C46H80NO8P
1,5	1,439	292,9	830,5699	829,5678	[M+H]+, [M+K]+, [M+Na]+, [M+NH4]+	PC 18:2_22:6	C48H80NO8P

1,45	1,451	295,3	854,5692	853,562	[M+H]+	PC 20:4_22:6	C50H80NO8P
1,65	1,374	280,5	706,5379	705,5306	[M+H]+	PC 30:0	C38H76NO8P
1,51	1,374	280,4	730,5381	729,5312	[M+H]+, [M+Na]+	PC 32:2	C40H76NO8P
1,79	1,402	285,9	746,5698	745,5625	[M+H]+	PC 33:1	C41H80NO8P
1,62	1,385	282,5	744,5541	743,5468	[M+H]+	PC 33:2	C41H78NO8P
1,47	1,386	282,7	754,5387	753,5314	[M+H]+	PC 34:4	C42H76NO8P
2,02	1,433	292,1	774,6008	773,5936	[M+H]+	PC 35:1	C43H84NO8P
1,65	1,402	285,9	770,5702	769,5629	[M+H]+	PC 35:3	C43H80NO8P
1,59	1,402	285,7	768,5568	767,5495	[M+H]+	PC 35:4	C43H78NO8P
2,34	1,461	297,7	790,632	789,6247	[M+H]+	PC 36:0	C44H88NO8P
1,42	1,399	285,2	778,5386	777,5314	[M+H]+	PC 36:6	C44H76NO8P
2,08	1,451	295,5	800,6166	799,6093	[M+H]+	PC 37:2	C45H86NO8P
1,81	1,434	292,2	796,5852	795,5779	[M+H]+	PC 37:4	C45H82NO8P
1,53	1,416	288,6	792,554	791,5468	[M+H]+	PC 37:6	C45H78NO8P
2,04	1,465	298,4	824,6171	823,6098	[M+H]+	PC 39:4	C47H86NO8P
2,09	1,476	300,4	838,632	837,6247	[M+H]+	PC 40:4	C48H88NO8P
1,93	1,468	298,8	836,6159	835,6087	[M+H]+	PC 40:5	C48H86NO8P
1,66	1,451	295,4	832,5846	831,5778	[M+H]+, [M+Na]+	PC 40:7	C48H82NO8P
1,69	1,467	298,5	858,601	857,5937	[M+H]+	PC 42:8	C50H84NO8P

Supplemental Table 3.
List of identified lysophosphatidylcholines and phosphatidylcholines in pancreas extract. Abbreviations: RT, retention time; m/z meas, mass to charge measured; Mass meas., measured mass; CCS, cross collisional section; Mob., mobility; LPC, lysophosphatidylcholine; PC, phosphatidylcholine. Primary ion has been used for quantification

RT [min]	Mob. 1/K0	CCS (Å²)	m/z meas.	M meas.	Ions	Lipid	Molecular Formula
0,37	1,091	224,9	468,309	467,3017	[M+H]+	LPC 14:0	C22H46NO7P
0,48	1,128	232,1	496,3402	495,3329	[M+H]+	LPC 16:0	C24H50NO7P
0,38	1,105	227,5	494,3246	493,3173	[M+H]+	LPC 16:1	C24H48NO7P
0,54	1,148	236,1	510,3556	509,3484	[M+H]+, [M+Na]+	LPC 17:0	C25H52NO7P
0,64	1,165	239,4	524,3713	523,3641	[M+H]+	LPC 18:0	C26H54NO7P
0,48	1,149	236,2	522,3557	521,3484	[M+H]+	LPC 18:1	C26H52NO7P
0,42	1,12	230,3	520,3402	519,333	[M+H]+, [M+K]+, [M+Na]+	LPC 18:2	C26H50NO7P
0,36	1,107	227,5	518,3244	517,3171	[M+H]+	LPC 18:3	C26H48NO7P
0,75	1,181	242,6	538,3871	537,3798	[M+H]+	LPC 19:0	C27H56NO7P
0,54	1,166	239,5	536,3717	535,3644	[M+H]+	LPC 19:1	C27H54NO7P
0,88	1,203	246,9	552,4021	551,395	[M+H]+, [M+Na]+	LPC 20:0	C28H58NO7P
0,65	1,18	242,2	550,3865	549,3795	[M+H]+, [M+Na]+	LPC 20:1	C28H56NO7P

0,51	1,159	238	548,371	547,3637	[M+H]+	LPC 20:2	C28H54NO7P
0,44	1,146	235,2	546,3546	545,3475	[M+H]+, [M+K]+	LPC 20:3	C28H52NO7P
0,39	1,139	233,9	544,34	543,3327	[M+H]+	LPC 20:4	C28H50NO7P
0,34	1,123	230,6	542,3243	541,317	[M+H]+, [M+Na]+, [M+K]+	LPC 20:5	C28H48NO7P
1,15	1,238	253,8	580,4335	579,4265	[M+H]+, [M+Na]+	LPC 22:0	C30H62NO7P
0,91	1,213	248,7	578,4183	577,411	[M+H]+	LPC 22:1	C30H60NO7P
0,69	1,208	247,7	576,403	575,3958	[M+H]+	LPC 22:2	C30H58NO7P
0,47	1,172	240,4	572,3709	571,3636	[M+H]+	LPC 22:4	C30H54NO7P
0,43	1,161	238,2	570,3552	569,348	[M+H]+	LPC 22:5	C30H52NO7P
0,37	1,157	237,4	568,3397	567,3324	[M+H]+	LPC 22:6	C30H50NO7P
1,26	1,255	257,1	594,4496	593,4423	[M+H]+	LPC 23:0	C31H64NO7P
1,39	1,273	260,8	608,4646	607,4576	[M+H]+, [M+Na]+, [M+K]+	LPC 24:0	C32H66NO7P
1,16	1,247	255,5	606,4492	605,4421	[M+H]+, [M+Na]+	LPC 24:1	C32H64NO7P
0,97	1,228	251,5	604,4337	603,4264	[M+H]+	LPC 24:2	C32H62NO7P
1,39	1,282	262,2	634,4806	633,4733	[M+H]+	LPC 26:1	C34H68NO7P
1,52	1,37	279,6	730,5381	729,5314	[M+H]+, [M+K]+, [M+Na]+	PC 14:0_18:2	C40H76NO8P
1,38	1,362	278	728,5226	727,5153	[M+H]+	PC 14:0_18:3	C40H74NO8P
1,48	1,386	282,6	754,5377	753,5304	[M+H]+	PC 14:0_20:4	C42H76NO8P
1,34	1,373	280,1	752,5221	751,5157	[M+H]+, [M+Na]+	PC 14:0_20:5	C42H74NO8P
1,43	1,402	285,8	778,5381	777,5308	[M+H]+	PC 14:0_22:6	C44H76NO8P
1,78	1,388	283,3	720,5539	719,5466	[M+H]+	PC 15:0_16:0	C39H78NO8P
1,89	1,407	287,1	734,5707	733,5634	[M+H]+	PC 16:0_16:0	C40H80NO8P
1,69	1,387	282,9	732,5538	731,5465	[M+H]+	PC 16:0_16:1	C40H78NO8P
1,63	1,382	281,9	744,5539	743,5466	[M+H]+	PC 16:0_17:2	C41H78NO8P
2,13	1,435	292,7	762,6013	761,5941	[M+H]+	PC 16:0_18:0	C42H84NO8P
1,92	1,423	290,1	760,5855	759,5782	[M+H]+	PC 16:0_18:1	C42H82NO8P
1,74	1,408	287,1	758,5689	757,5616	[M+H]+	PC 16:0_18:2	C42H80NO8P
1,62	1,395	284,5	756,5535	755,5462	[M+H]+	PC 16:0_18:3	C42H78NO8P
1,7	1,423	290	782,5683	781,5611	[M+H]+	PC 16:0_20:4	C44H80NO8P
1,56	1,405	286,3	780,555	779,5477	[M+H]+	PC 16:0_20:5	C44H78NO8P
1,72	1,445	294,4	808,5844	807,5771	[M+H]+	PC 16:0_22:5	C46H82NO8P
1,65	1,432	291,7	806,57	805,5628	[M+H]+	PC 16:0_22:6	C46H80NO8P
1,86	1,418	289,2	772,5845	771,5773	[M+H]+	PC 17:0_18:2	C43H82NO8P
1,83	1,437	292,8	796,5849	795,5776	[M+H]+	PC 17:0_20:4	C45H82NO8P
2,16	1,448	295	788,617	787,6097	[M+H]+	PC 18:0_18:1	C44H86NO8P
1,98	1,437	292,8	786,6012	785,5939	[M+H]+	PC 18:0_18:2	C44H84NO8P
2,19	1,465	298,3	814,631	813,6246	[M+H]+, [M+Na]+, [M+K]+	PC 18:0_20:2	C46H88NO8P

2,03	1,458	296,9	812,6168	811,6091	[M+H]+, [M+H-H2O]+	PC 18:0_20:3	C46H86NO8P
1,94	1,455	296,4	810,6015	809,5942	[M+H]+	PC 18:0_20:4	C46H84NO8P
2,11	1,482	301,6	838,6313	837,624	[M+H]+	PC 18:0_22:4	C48H88NO8P
1,88	1,464	298,1	834,6003	833,5931	[M+H]+	PC 18:0_22:6	C48H84NO8P
1,79	1,426	290,6	784,5858	783,5785	[M+H]+	PC 18:1_18:2	C44H82NO8P
1,67	1,452	295,5	832,5846	831,5773	[M+H]+	PC 18:1_22:6	C48H82NO8P
1,56	1,425	290,2	806,5692	805,5619	[M+H]+	PC 18:2_20:4	C46H80NO8P
1,44	1,415	288,2	804,5533	803,5461	[M+H]+	PC 18:2_20:5	C46H78NO8P
1,51	1,439	293	830,5702	829,563	[M+H]+	PC 18:2_22:6	C48H80NO8P
2,06	1,467	298,8	824,6161	823,6088	[M+H]+	PC 19:0_20:4	C47H86NO8P
1,84	1,453	296	822,6013	821,594	[M+H]+	PC 19:1_20:4	C47H84NO8P
1,95	1,474	300,1	836,6161	835,6089	[M+H]+	PC 20:1_20:4	C48H86NO8P
1,77	1,455	296,3	834,6011	833,5935	[M+H]+, [M+H-H2O]+	PC 20:2_20:4	C48H84NO8P
1,38	1,428	290,7	828,5532	827,546	[M+H]+	PC 20:4_20:5	C48H78NO8P
1,46	1,449	294,9	854,5689	853,5616	[M+H]+	PC 20:4_22:6	C50H80NO8P
2,59	1,502	305,7	844,6792	843,6719	[M+H]+	PC 22:0_18:1	C48H94NO8P
2,4	1,51	307,1	866,6632	865,6559	[M+H]+	PC 22:0_20:4	C50H92NO8P
2,21	1,484	302,1	840,6466	839,6393	[M+H]+	PC 22:1_18:2	C48H90NO8P
2,59	1,525	310,2	870,695	869,6877	[M+H]+	PC 24:0_18:2	C50H96NO8P
2,1	1,495	304,2	864,6471	863,6398	[M+H]+	PC 24:2_18:3	C50H90NO8P
1,44	1,343	274,5	678,5069	677,4997	[M+H]+	PC 28:0	C36H72NO8P
1,66	1,373	280,4	706,5376	705,5304	[M+H]+	PC 30:0	C38H76NO8P
1,47	1,356	276,9	704,5221	703,5148	[M+H]+	PC 30:1	C38H74NO8P
1,47	1,365	278,6	728,5223	727,5151	[M+H]+	PC 32:3	C40H74NO8P
1,81	1,402	286	746,5696	745,5623	[M+H]+	PC 33:1	C41H80NO8P
2,04	1,433	292,1	774,6002	773,5929	[M+H]+	PC 35:1	C43H84NO8P
1,6	1,4	285,5	768,5539	767,5466	[M+H]+	PC 35:4	C43H78NO8P
1,45	1,392	283,7	766,5385	765,5312	[M+H]+	PC 35:5	C43H76NO8P
2,36	1,458	297,2	790,6318	789,6245	[M+H]+	PC 36:0	C44H88NO8P
2,27	1,461	297,6	802,6319	801,6246	[M+H]+	PC 37:1	C45H88NO8P
2,09	1,452	295,8	800,6159	799,6093	[M+H]+, [M+Na]+	PC 37:2	C45H86NO8P
2,58	1,487	302,8	818,6639	817,6566	[M+H]+	PC 38:0	C46H92NO8P
2,37	1,472	299,8	816,6478	815,6407	[M+H]+, [M+Na]+	PC 38:1	C46H90NO8P
1,85	1,448	294,9	810,6002	809,5929	[M+H]+	PC 38:4	C46H84NO8P
1,79	1,444	294,2	808,5844	807,5771	[M+H]+	PC 38:5	C46H82NO8P
2,32	1,477	300,6	828,647	827,6397	[M+H]+	PC 39:2	C47H90NO8P
2,77	1,519	309,1	846,6947	845,6874	[M+H]+	PC 40:0	C48H96NO8P
2,18	1,479	301,1	838,632	837,6247	[M+H]+	PC 40:4	C48H88NO8P
2,03	1,477	300,6	836,6159	835,6086	[M+H]+	PC 40:5	C48H86NO8P
1,51	1,493	304	830,5694	829,5621	[M+H]+	PC 40:8	C48H80NO8P
2,78	1,532	311,6	872,71	871,7028	[M+H]+	PC 42:1	C50H98NO8P
2,66	1,52	309,2	870,6945	869,687	[M+H]+, [M+Na]+	PC 42:2	C50H96NO8P

2,43	1,508	306,9	868,678	867,6708	[M+H]+	PC 42:3	C50H94NO8P
2,18	1,496	304,3	864,6478	863,6406	[M+H]+	PC 42:5	C50H90NO8P
2,11	1,495	304,1	862,6305	861,6232	[M+H]+	PC 42:6	C50H88NO8P
1,46	1,472	299,5	870,6008	869,5935	[M+H]+	PC 43:9	C51H84NO8P
2,61	1,536	312,3	894,6945	893,6872	[M+H]+	PC 44:4	C52H96NO8P
2,39	1,524	309,9	892,6785	891,6712	[M+H]+	PC 44:5	C52H94NO8P
2,21	1,512	307,5	890,6623	889,655	[M+H]+	PC 44:6	C52H92NO8P
2,56	1,549	314,8	918,695	917,6877	[M+H]+	PC 46:6	C54H96NO8P

Supplemental Table 4
List of identified lysophosphatidylcholines and phosphatidylcholines in liver extract. Abbreviations: RT, retention time; m/z meas, mass to charge measured; Mass meas., measured mass; CCS, cross collisional section; Mob., mobility; LPC, lysophosphatidylcholine; PC, phosphatidylcholine. Primary ion has been used for quantification

RT [min]	Mob. 1/K0	CCS (Å²)	m/z meas.	M meas.	Ions	Lipid	Molecular Formula
0,39	1,089	224,4	468,309	467,3017	[M+H]+	LPC 14:0	C22H46NO7P
0,43	1,107	228	482,3246	481,3173	[M+H]+	LPC 15:0	C23H48NO7P
0,49	1,128	232,1	496,3401	495,3328	[M+H]+	LPC 16:0	C24H50NO7P
0,4	1,104	227,3	494,3241	493,3172	[M+H]+, [M+K]+, [M+Na]+	LPC 16:1	C24H48NO7P
0,55	1,148	236,2	510,3553	509,3483	[M+H]+, [M+Na]+	LPC 17:0	C25H52NO7P
0,44	1,125	231,4	508,3404	507,3331	[M+H]+	LPC 17:1	C25H50NO7P
0,64	1,166	239,6	524,3712	523,3639	[M+H]+	LPC 18:0	C26H54NO7P
0,5	1,146	235,5	522,3551	521,3479	[M+H]+	LPC 18:1	C26H52NO7P
0,41	1,12	230,3	520,34	519,3328	[M+H]+	LPC 18:2	C26H50NO7P
0,38	1,107	227,6	518,3245	517,3172	[M+H]+	LPC 18:3	C26H48NO7P
0,75	1,181	242,6	538,3866	537,3793	[M+H]+	LPC 19:0	C27H56NO7P
0,89	1,203	247	552,4022	551,3955	[M+H]+, [M+Na]+, [M+K]+	LPC 20:0	C28H58NO7P
0,67	1,179	241,9	550,3867	549,3795	[M+H]+, [M+Na]+	LPC 20:1	C28H56NO7P
0,4	1,138	233,7	544,34	543,3327	[M+H]+	LPC 20:4	C28H50NO7P
1,15	1,238	253,9	580,4333	579,4264	[M+H]+, [M+Na]+	LPC 22:0	C30H62NO7P
0,91	1,214	248,8	578,4183	577,411	[M+H]+	LPC 22:1	C30H60NO7P
0,39	1,154	236,7	568,3398	567,3326	[M+H]+, [M+K]+, [M+Na]+	LPC 22:6	C30H50NO7P
1,26	1,255	257,2	594,4497	593,4424	[M+H]+	LPC 23:0	C31H64NO7P
1,39	1,272	260,6	608,4645	607,4574	[M+H]+, [M+Na]+	LPC 24:0	C32H66NO7P

1,16	1,248	255,5	606,4494	605,4421	[M+H]+	LPC 24:1	C32H64NO7P
1,4	1,365	278,5	728,5223	727,5157	[M+H]+, [M+Na]+	PC 14:0_18:3	C40H74NO8P
1,48	1,384	282,2	754,5377	753,5304	[M+H]+	PC 14:0_20:4	C42H76NO8P
1,34	1,374	280,2	752,5222	751,5155	[M+H]+, [M+Na]+	PC 14:0_20:5	C42H74NO8P
1,43	1,399	285,2	778,5388	777,5317	[M+H]+, [M+Na]+, [M+K]+	PC 14:0_22:6	C44H76NO8P
1,6	1,4	285,3	768,5538	767,5465	[M+H]+	PC 15:0_20:4	C43H78NO8P
1,89	1,404	286,4	734,5696	733,5624	[M+H]+, [M+Na]+	PC 16:0_16:0	C40H80NO8P
1,69	1,387	283	732,5537	731,5464	[M+H]+	PC 16:0_16:1	C40H78NO8P
1,81	1,402	285,9	746,569	745,5617	[M+H]+	PC 16:0_17:1	C41H80NO8P
2,13	1,434	292,4	762,6009	761,5936	[M+H]+	PC 16:0_18:0	C42H84NO8P
1,92	1,422	289,9	760,5857	759,5784	[M+H]+	PC 16:0_18:1	C42H82NO8P
1,74	1,407	286,8	758,5696	757,5623	[M+H]+	PC 16:0_18:2	C42H80NO8P
1,62	1,396	284,6	756,5539	755,5467	[M+H]+	PC 16:0_18:3	C42H78NO8P
1,71	1,422	289,8	782,5693	781,5621	[M+H]+	PC 16:0_20:4	C44H80NO8P
1,65	1,434	292,1	806,5691	805,5618	[M+H]+	PC 16:0_22:6	C46H80NO8P
1,51	1,37	279,5	730,5378	729,5305	[M+H]+	PC 16:1_16:1	C40H76NO8P
1,46	1,418	288,9	804,5547	803,5474	[M+H]+	PC 16:1_22:6	C46H78NO8P
2,04	1,431	291,8	774,6006	773,5933	[M+H]+	PC 17:0_18:1	C43H84NO8P
1,76	1,451	295,4	820,5847	819,5775	[M+H]+	PC 17:0_22:6	C47H82NO8P
1,86	1,417	288,8	772,5847	771,5774	[M+H]+	PC 17:1_18:1	C43H82NO8P
1,57	1,438	292,9	818,5695	817,5622	[M+H]+	PC 17:1_22:6	C47H80NO8P
2,16	1,448	295,1	788,6172	787,6099	[M+H]+	PC 18:0_18:1	C44H86NO8P
1,98	1,436	292,7	786,6016	785,5944	[M+H]+	PC 18:0_18:2	C44H84NO8P
2,03	1,456	296,5	812,6171	811,6098	[M+H]+	PC 18:0_20:3	C46H86NO8P
1,94	1,454	296,1	810,6009	809,5936	[M+H]+	PC 18:0_20:4	C46H84NO8P
2,11	1,48	301,4	838,6314	837,6236	[M+H]+, [M+K]+, [M+Na]+	PC 18:0_22:4	C48H88NO8P
2,03	1,476	300,5	836,6159	835,6086	[M+H]+	PC 18:0_22:5	C48H86NO8P
1,88	1,463	297,8	834,6016	833,5943	[M+H]+	PC 18:0_22:6	C48H84NO8P
2,29	1,502	305,7	866,6624	865,6551	[M+H]+	PC 18:0_24:4	C50H92NO8P
1,8	1,424	290,3	784,5858	783,5785	[M+H]+	PC 18:1_18:2	C44H82NO8P
1,73	1,443	294	808,5853	807,5781	[M+H]+	PC 18:1_20:4	C46H82NO8P
1,67	1,454	295,9	832,5856	831,5783	[M+H]+	PC 18:1_22:6	C48H82NO8P
1,51	1,439	293	830,5701	829,5629	[M+H]+	PC 18:2_22:6	C48H80NO8P
2,1	1,449	295,2	800,6158	799,6088	[M+H]+, [M+Na]+	PC 19:0_18:2	C45H86NO8P
2,26	1,484	302,1	840,6472	839,6399	[M+H]+	PC 20:0_20:3	C48H90NO8P
2,18	1,479	301,1	838,6315	837,6248	[M+H]+, [M+K]+, [M+Na]+	PC 20:0_20:4	C48H88NO8P
2,11	1,495	304,2	862,6314	861,6244	[M+H]+, [M+Na]+	PC 20:0_22:6	C50H88NO8P
1,95	1,475	300,2	836,6157	835,6084	[M+H]+	PC 20:1_20:4	C48H86NO8P

1,89	1,483	301,8	860,6165	859,6092	[M+H]+	PC 20:1_22:6	C50H86NO8P
1,46	1,452	295,5	854,5696	853,5623	[M+H]+	PC 20:4_22:6	C50H80NO8P
0,8	1,235	253	594,4133	593,4061	[M+H]+	PC 22:0	C30H60NO8P
2,44	1,509	307,1	868,6787	867,6714	[M+H]+	PC 22:0_20:3	C50H94NO8P
2,4	1,523	309,7	892,6791	891,6718	[M+H]+	PC 22:0_22:5	C52H94NO8P
2,62	1,536	312,4	894,6944	893,6871	[M+H]+	PC 24:0_20:4	C52H96NO8P
1,44	1,343	274,4	678,5072	677,4999	[M+H]+	PC 28:0	C36H72NO8P
1,66	1,374	280,5	706,5383	705,531	[M+H]+	PC 30:0	C38H76NO8P
1,47	1,356	276,8	704,5226	703,5153	[M+H]+	PC 30:1	C38H74NO8P
1,3	1,343	274,2	702,5068	701,4995	[M+H]+	PC 30:2	C38H72NO8P
1,41	1,356	276,8	716,5233	715,5161	[M+H]+	PC 31:2	C39H74NO8P
1,27	1,355	276,4	726,507	725,4998	[M+H]+	PC 32:4	C40H72NO8P
1,63	1,383	282,1	744,5534	743,5461	[M+H]+	PC 33:2	C41H78NO8P
1,38	1,371	279,7	740,526	739,5187	[M+H]+	PC 33:4	C41H74NO8P
2,24	1,448	295,1	776,6166	775,6093	[M+H]+	PC 35:0	C43H86NO8P
1,67	1,403	286	770,5695	769,5622	[M+H]+	PC 35:3	C43H80NO8P
1,45	1,387	282,8	766,5384	765,5311	[M+H]+	PC 35:5	C43H76NO8P
2,36	1,459	297,3	790,6323	789,625	[M+H]+	PC 36:0	C44H88NO8P
1,3	1,389	283,1	776,5227	775,5154	[M+H]+	PC 36:7	C44H74NO8P
2,27	1,461	297,6	802,6318	801,6255	[M+H]+, [M+Na]+	PC 37:1	C45H88NO8P
1,83	1,435	292,4	796,5844	795,5771	[M+H]+	PC 37:4	C45H82NO8P
1,62	1,42	289,3	794,5697	793,5624	[M+H]+	PC 37:5	C45H80NO8P
1,54	1,416	288,6	792,5535	791,5462	[M+H]+	PC 37:6	C45H78NO8P
2,58	1,487	302,9	818,6634	817,6561	[M+H]+	PC 38:0	C46H92NO8P
2,38	1,472	299,8	816,6474	815,6401	[M+H]+, [M+Na]+	PC 38:1	C46H90NO8P
2,21	1,463	298,1	814,6324	813,6252	[M+H]+	PC 38:2	C46H88NO8P
1,79	1,445	294,3	808,5845	807,5772	[M+H]+	PC 38:5	C46H82NO8P
1,36	1,409	287	802,5376	801,5315	[M+H]+, [M+Na]+	PC 38:8	C46H76NO8P
2,06	1,466	298,5	824,6162	823,6089	[M+H]+	PC 39:4	C47H86NO8P
1,84	1,453	295,8	822,6009	821,5936	[M+H]+	PC 39:5	C47H84NO8P
2,77	1,517	308,8	846,6953	845,688	[M+H]+	PC 40:0	C48H96NO8P
2,59	1,501	305,5	844,679	843,6718	[M+H]+	PC 40:1	C48H94NO8P
1,25	1,417	288,5	826,5387	825,5314	[M+H]+	PC 40:10	C48H76NO8P
1,38	1,428	290,7	828,5532	827,5471	[M+H]+, [M+Na]+	PC 40:9	C48H78NO8P
2,78	1,531	311,5	872,7114	871,7041	[M+H]+	PC 42:1	C50H98NO8P
2,65	1,52	309,2	870,6945	869,6872	[M+H]+	PC 42:2	C50H96NO8P
2,4	1,509	307,1	866,6628	865,6555	[M+H]+	PC 42:4	C50H92NO8P
2,18	1,495	304,3	864,6476	863,6403	[M+H]+	PC 42:5	C50H90NO8P
1,69	1,468	298,8	858,6007	857,5934	[M+H]+	PC 42:8	C50H84NO8P
1,56	1,457	296,4	856,585	855,5777	[M+H]+	PC 42:9	C50H82NO8P
